# Exploring motor unit and neuromuscular junction dysfunction in aging and sarcopenia: insights from electromyography in systematic review

**DOI:** 10.1007/s11357-025-01760-0

**Published:** 2025-07-08

**Authors:** Can Cui, Yong Hu, Ronald Man Yeung Wong, Ning Zhang, Yuzhou Guan, Wing-hoi Cheung

**Affiliations:** 1 Musculoskeletal Research Laboratory, Department of Orthopaedics & Traumatology, The Chinese University of Hong Kong, Hong Kong SAR, China; 2 Li Ka Shing Institute of Health Sciences, The Chinese University of Hong Kong, Hong Kong SAR, China; 3 Department of Orthopaedics & Traumatology, School of Clinical Medicine, Faculty of Medicine, The University of Hong Kong, Li Kai Shing, Hong Kong SAR, China; 4 Department of Neurology, Peking Union Medical College Hospital, Chinese Academy of Medical Sciences, Beijing, China

**Keywords:** Neuromuscular junction, Aging, Electromyography, Sarcopenia, Motor unit

## Abstract

The neuromuscular junction (NMJ) is a vital interface between motor neurons and muscle fibers, and alterations in its structure and function can substantially influence the onset and progression of sarcopenia. Electromyography (EMG) is a critical tool to assess motor unit and NMJ function, providing insights into neuromuscular activation patterns and the integrity of motor unit communication. However, its implications for aging and muscle performance during sarcopenia have not been fully discussed. Therefore, we conducted a systematic review using the PubMed, Embase, and Web of Science databases by employing relevant keywords. A total of 53 articles were included. This review explored the various alterations in the NMJ associated with aging, their functional implications, and potential interventions to mitigate these effects, highlighting the structural and functional alterations of the NMJ during aging and sarcopenia. Key findings include early NMJ transmission instability, motor unit loss and compensatory remodeling, and impaired neuromuscular activation preceding overt muscle atrophy and weakness. Notably, biomarkers such as C-terminal agrin fragment and neurofilament light chain, along with EMG-derived parameters (e.g., jitter, jiggle, MUNE), are sensitive indicators of NMJ deterioration. Both physical inactivity and hormonal changes (e.g., menopause) accelerate NMJ decline, while interventions such as resistance and endurance training, nutritional supplementation, and emerging gene therapies demonstrate potential to preserve or restore NMJ structure and function. In conclusion, this systematic review underscores the importance of NMJ dysfunction in aging and sarcopenia, advocating further research into diagnostic biomarkers and therapeutic strategies to enhance NMJ integrity. The interplay between aging, exercise, and NMJ function is complex and requires a nuanced approach to rehabilitation and exercise strategies tailored to the aging population. Future directions should prioritize the development of sensitive biomarkers, mechanistic studies of NMJ degeneration, and rigorous evaluation of multimodal interventions to mitigate neuromuscular decline and promote healthy aging.

**Graphical Abstract:**

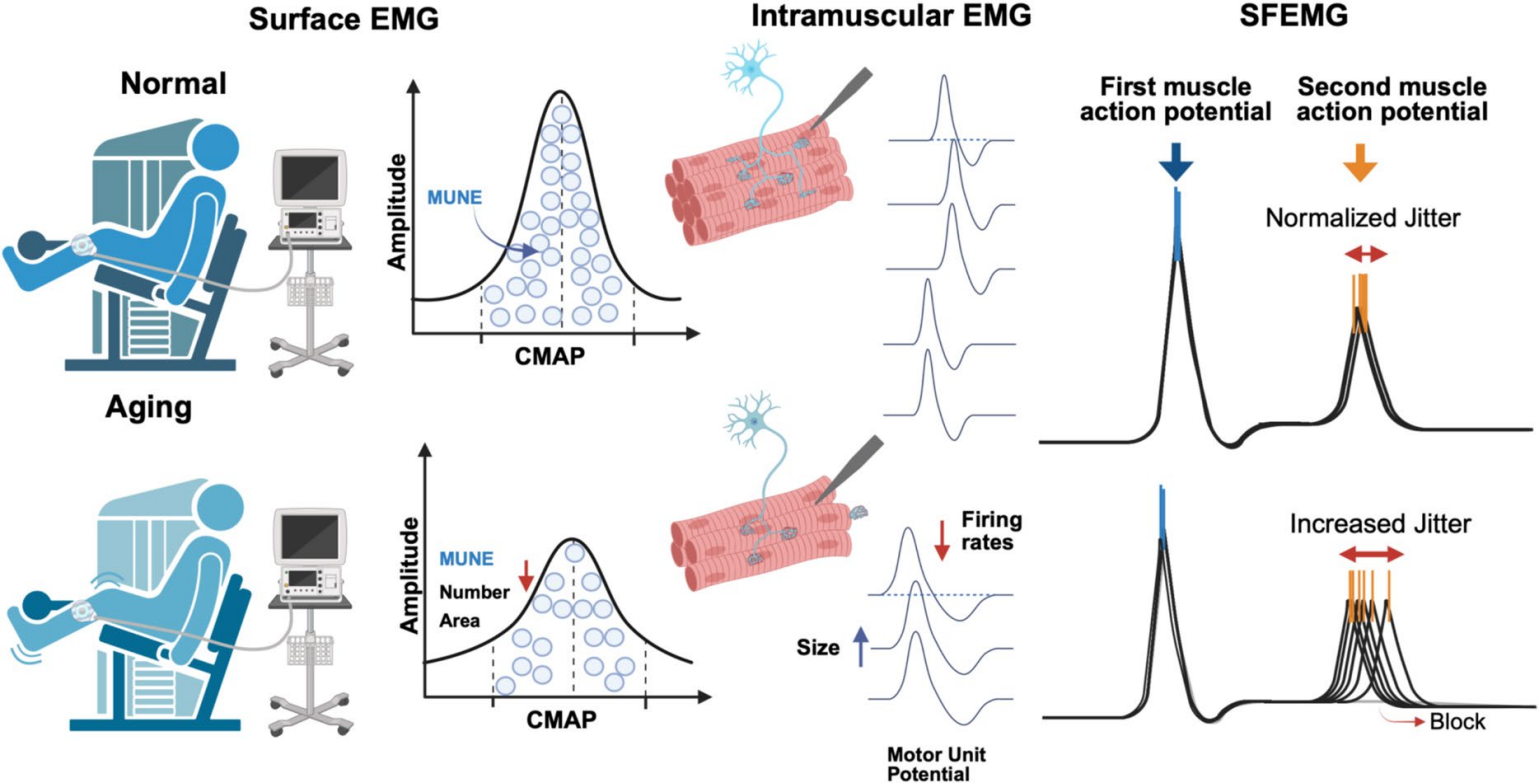

## Introduction

Aging is commonly associated with a decline in skeletal muscle function; however, it is crucial to distinguish between the normal, age-related changes in muscle and the pathological condition known as sarcopenia. Sarcopenia is characterized by a progressive loss of muscle mass and strength, leading to increased frailty, reduced mobility, and a higher risk of falls and fractures among older adults. This decline in muscle function can severely affect an individual’s quality of life and independence, resulting in more significant healthcare costs and a heightened burden on health systems globally.

Although the multifactorial nature of sarcopenia has been well documented, comprising factors such as hormonal changes, inflammation, nutritional deficiencies, and sedentary lifestyles, there is an increasing number of research focusing on the role of neuromuscular junction (NMJ) dysfunction as a critical factor contributing to this condition [1, 2]. NMJ is a vital interface between motor neurons and muscle fibers, facilitating the transmission of signals that initiate muscle contraction. At this junction, communication between the nervous and muscular systems occurs, making its integrity essential for optimal muscle function. Alterations in the NMJ’s structure and function, including changes in neurotransmitter release, receptor density, and synaptic architecture, can substantially influence the onset and progression of sarcopenia. Age-related degeneration in NMJ could lead to impaired neuromuscular transmission and weakened muscle contraction capabilities.

Electromyography (EMG), a cornerstone in neuromuscular research, encompasses a range of methodologies to assess muscle activation and NMJ integrity. The reviewed studies in this paper utilized several EMG techniques, including surface EMG, needle EMG, and single-fiber EMG (SFEMG), each offering distinct advantages and diagnostic sensitivities. Surface EMG employs electrodes placed on the skin to non-invasively record the summated electrical activity of underlying muscle groups, making it ideal for large-scale assessments of voluntary muscle activation and motor unit recruitment patterns [3]. In contrast, needle EMG involves the insertion of fine electrodes directly into the muscle tissue, enabling the analysis of individual motor unit potentials (MUPs), motor unit firing rates, and more precise characterization of neuromuscular transmission abnormalities. Single-fiber EMG, a specialized needle technique, isolates action potentials from individual muscle fibers, allowing for detailed quantification of NMJ transmission variability through parameters such as jitter and blocking, which are sensitive markers for early NMJ dysfunction [4]. High-density and multi-channel EMG, also referenced in the literature, further enhance spatial resolution and the ability to dissect complex motor unit behaviors. The selection of EMG modality is guided by the specific research or clinical objective: while surface EMG is suitable for non-invasive, population-level studies, needle EMG and SFEMG are indispensable for the early detection and detailed analysis of NMJ pathology, as demonstrated across the included studies.

This systematic review aims to explore the various alterations at the motor unit and NMJ associated with aging, their functional implications, and potential interventions to mitigate these effects. By thoroughly examining relevant studies published in the literature, we highlight the critical importance of NMJ integrity in maintaining optimal muscle performance during aging. The review will delve into the specific changes that occur at the NMJ with age, such as alterations in motor unit characteristics, including increased motor unit potential sizes and decreased firing rates, which are often assessed through electromyography (EMG). Additionally, we will discuss how EMG measures can provide insights into neuromuscular transmission efficiency, revealing changes in the rate of neuromuscular activation and muscle recruitment patterns that correlate with overall muscle strength and functionality. These EMG-derived parameters are essential for understanding the impact of aging on neuromuscular control, allowing for the identification of potential therapeutic strategies to enhance NMJ health and muscle performance in older adults.

## Methods

### Search strategy

A systematic literature search was conducted on 20 May 2025 across PubMed, Embase, and Web of Science databases to identify relevant publications and extract corresponding results. The search strategy employed the following keywords: (ageing or aging or aged or aged) AND (Neuromuscular Junction) AND (Motor Function). Additionally, the reference lists of included studies and reviews were manually scrutinized for further relevant studies. The study adhered to the PRISMA guidelines.

### Selection criteria

Inclusion criteria included the following studies: (1) focused on electromyography, (2) focused on functional and morphological changes of motor unit and NMJ during normal aging, (3) with full-text articles published in English.

Exclusion criteria were as follows: (1) irrelevance to aging; (2) irrelevance to electromyography; (3) studies on neuromuscular disorders, e.g., motoneuron diseases (MNDs) such as amyotrophic lateral sclerosis (ALS) and myasthenia gravis; (4) review articles; (5) reports published solely as conference abstracts.

### Study selection

Study selection and data extraction were independently conducted by two reviewers. The initial screening involved the exclusion of irrelevant papers based on their titles and abstracts. Duplicate citations were identified and eliminated using the Endnote reference manager. The remaining potentially relevant articles were assessed according to inclusion and exclusion criteria. Any disagreements were resolved by discussion with a third reviewer.

### Data extraction

The following information was extracted by two independent reviewers: author, year of publication, animal model, age range, methodology, muscle types, morphological results, functional results, EMG results, and other related data concerning motor units and NMJ alternations. All studies that met the pre-defined criteria were included in the analysis.

### Data analysis

Given that this study included 25 clinical studies and 28 animal studies incorporated diverse animal models and methodologies, the data exhibited high heterogeneity.

## Results

**Fig. 1:**
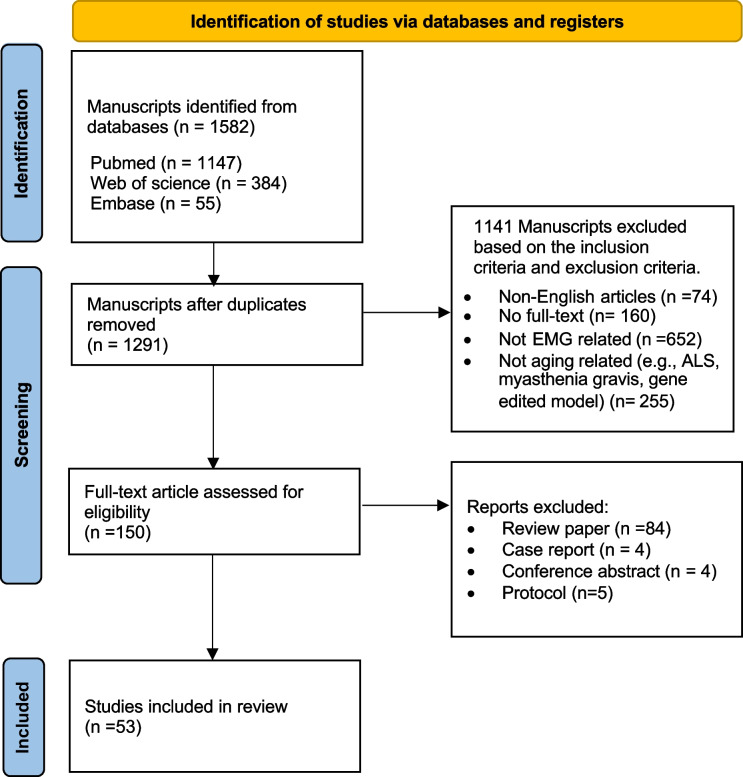
Flow diagram for the literature selection process. Total 53 studies met inclusion

The 53 selected studies were conducted between 1964 and 2025. Of these, 25 utilized human subjects, 20 utilized mice, 2 utilized Drosophila, and 6 involved a rat model. The flowchart detailing the selection process is shown in Fig. 1. The characteristics of the included studies are shown in Tables 1, 2, and 3.

**Table 1:** Characteristics of clinical studies

Study	Subject	Gender	Age	Muscle	Muscle performance measurements	Muscle mass measurements	Neuromuscular activation measurements	Muscle related results	Neurophysiological-related results	Conclusion
**Lamoureux et al. **[5]** (2001)**	Human	Male and female	63.3 ± 2.6 years 75 ± 3.9 years	Gluteus maximus Iliopsoas Adductor magnus Gluteus medius Quadriceps Hamstrings Gastrocnemius	• Isometric strength testing: Maximal voluntary isometric contraction (MVC) and maximal isometric rate of torque development (MaxRTD) • Dynamic isotonic strength testing by one-repetition maximum (1-RM) exercises: leg extension, leg curl, calf raise, hip flexion, hip extension, hip adduction, hip abduction	Dual-energy x-ray absorptiometry (DXA): Bone-free lean tissue mass (BFLT)	Surface electromyographic (EMG) activity on rectus femoris (RF), vastus lateralis (VL), and vastus medialis (VM): integrated electromyographic activity (iEMG)–time curves (expressed as a percentage of peak iEMG)	*Old vs Older* *Muscle performance* *In old group* • Greater MVC and normalized MVC by body mass of BFLT mass • Stronger 1-RM scores and significant percentage differences • Greater isometric MaxRTD • Shorter time to move between torques in the absolute torque-time curves of the knee extensor muscles *Muscle mass* • Greater mean BFLT mass value of the thigh muscle • No significant differences in the mean percentage body fat value	*Old vs older* *In old group* • Significantly higher iEMG of VL, RF, and VM at each 100-ms epoch from the start of muscle activation up to 500 ms	• Worsening of the neuromuscular capacity of older adults compared with old adults • Maximal strength measures deteriorate with aging with an accelerated loss of dynamic strength • Declines in strength appear to be related to a reduction in lean tissue mass • The explosive capacity of the older adults decreases with age
**Piasecki et al. **[19]** (2016)**	Human	Male	25.3 ± 4.8 years 71.4 ± 6.2 years	Quadriceps Vastus lateralis (VL)	• Grip strength • Knee extension maximal voluntary contraction (MVC) • The Short Physical Performance Battery (SPPB)	• Dual-energy X-ray absorptiometry: fat and muscle mass • Magnetic resonance imaging (MRI): Quadriceps and vastus lateralis CSA	• Surface EMG: compound muscle action potential (CMAP) and motor unit number estimates (MUNEs) • Intramuscular (I.M.) EMG: motor unit potential (MUP) size, firing rates, phases, turns and near fiber (NF) jiggle	*Young vs old* *Muscle performance* *In young group:* • Significantly lower body fat percentage • Significantly higher grip strength (left and right) • Significantly higher knee extension MVC *Muscle mass* • Significantly higher appendicular lean mass divided by height-squared (ALM/h^2^) • Significantly higher quadriceps and VL CSA • No significance in anterior thigh fat thickness and femur CSA	*Young vs old* • Compared to young MUs, older MUs had larger MUPs with more phases and turns, lower fireing rates, increased fiber count and NF jiggle • Significantly larger CMAP in younger participants • Significant higher MUNE values in younger group • The MUNE values were significantly correlated with quadriceps CSA • Surface-recorded, ensemble-averaged sMUPs did not differ between young and older participants	*MU remodeling during healthy aging* • Reduction in the total number of MUs • Increase in the size of the surviving MUs • Increased instability in NMJ transmission • Decrease in surviving MU firing rates
**Kwon et al. **[11]** (2014)**	Human	Female	25.1 ± 3.9 years 71.5 ± 4.8 years	Biceps brachii and triceps brachii muscles for the elbow Tibialis anterior and soleus muscles for the ankle	Goal-directed movements of the left elbow and left ankle: • Maximal voluntary contraction (MVC) tasks • Practice of three to five goal-directed movement trials at a different target from the actual target • 100 goal-directed movement trials either with the elbow or with ankle • Repeat MVC forces to access the level of fatigue • Repeat 1 through 4 with the elbow or ankle depending on which joint was used for the first day (counterbalanced)	N/A	Agonist and antagonist surface EMG activity during elbow flexion and the ankle dorsiflexion: EMG burst duration, amplitude, viability, and EMG coactivation	*Young vs older* • No significant difference in MVC force for the elbow flexion and the ankle dorsiflexion between • Similar pre- and post-MVC forces for the elbow and for the ankle between two groups • Greater endpoint error, position variability, and time variability with both the elbow flexion and ankle dorsiflexion tasks in older group	*Young vs older* • EMG burst was significantly greater for older adults compared with young adults for both the elbow flexion and ankle dorsiflexion • EMG burst variability was significantly lower for older adults for the elbow flexion and the ankle dorsiflexion • EMG burst coactivation was significantly smaller for older adults for elbow flexion whereas • EMG burst time overlap coactivation was smaller for older adults for ankle dorsiflexion *Correlation analysis* • Endpoint error during the ankle dorsiflexion task was significantly associated only with EMG burst • Endpoint error during the elbow flexion task was significantly associated only with EMG burst coactivation • Time variability during the ankle dorsi-flexion task was significantly associated only with the EMG burst • Time variability during the elbow flexion task was significantly associated with the EMG burst and EMG burst coactivation	• Age-associated impairments in endpoint control during goal-directed movements with the upper and lower limbs are related to an altered neuromuscular activation of the involved muscles • Greater endpoint control with the upper limb relative to the lower limb also is related to an altered neuromuscular activation of the involved muscles
**Watanabe et al. **[17]** (2020)**	Human	Male and female	73.1 ± 5.9 years	Knee extensor muscles Vastus lateralis (VL)	• Isometric knee extension of the right leg: MVC and rate of force development (RFD) • Isometric knee extension ramp contractions from 0 to 30% of MVC (Ramp30) and 0 to 70% of MVC (Ramp70)	• Body impedance measurements: Body mass, whole body muscle mass, and lower limb muscle mass • Ultrasonographic imaging: Thickness of knee extensor muscles of the right leg	Multi-channel surface EMG from the VL muscle of the right leg during Ramp 30 and Ramp70: Firing rates of individual motor units	*Placebo (PLA) vs MFGM* *Both group with resistance training* • No significant differences in body mass, whole body muscle mass, lower limb muscle mass, muscle tissue thicknesses, MVC, or RFD between MFGM and PLA groups	*Placebo (PLA) vs MFGM* *Both group with resistance training* • MFGM group showed greater firing rates than the PLA group after 2 weeks of intervention for Rmap30 and Ramp70 • The numbers of pairs of motor unit groups showing a significant difference increased from 0 to 2 weeks in the MFGM group and from 2 to 4 weeks in the PLA group for both Ramp 30 and Ramp70	Motor unit adaptation following resistance training is modulated by MFGM supplementation in older adults
**Clark et al. **[6]** (2011)**	Human	Male and female	47.5 ± 4.6 years 73.9 ± 3.5 years 77.4 ± 4.8 years	Rectus femoris (RF) Vastus medialis (VM) Vastus lateralis (VL)	• Isometric maximal voluntary contraction (MVC) by one-repetition maximum (1-RM) method • Maximal-effort trials at each of two resistance levels, 260 N and 70%1RM (5 times): Acceleration and power	N/A	Surface EMG: amplitude, pre-movement time (duration between EMG onset and movement onset) and the rate of EMG rise	*Middle-aged healthy adult (MH) vs older healthy adults (OH) vs older adults with mobility limitations (OML)* • Significantly less force in OML group at 1-RM and 70% 1-RM than both MH and OH • Significantly higher proportion of 1-RM strength for OML at 260 N resistance condition than both MH and OH • Significantly lower leg press acceleration and leg press power in OML relative to MH and OH at 260 N and at 70% 1RM	*Middle-aged healthy adult (MH) vs older healthy adults (OH) vs older adults with mobility limitations (OML)* • Non-normalized EMG amplitude (mV) during the isometric MVC did not significantly differ between the three experimental groups • Significantly lower pre-movement time and rate of EMG rise.in OML compared with both MH • and OH at 260 N and at 70% 1RM *Correlation Analysis* • Across all older participants, rate of EMG rise was positively associated with acceleration and power	Slowing of neuromuscular activation rate is associated with compromised dynamic muscle performance
**Clark et al. **[7]** (2013)**	Human	Male and female	70.8 ± 4.5 years 71.4 ± 5.0 years	Triceps surae muscles: soleus (SO) and medial gastrocnemius (MG) Quadriceps Hamstrings	• 10-m walking speed (usual and maximum) and 400 m usual speed • Plantarflexion force assessed with a rapid bilateral heel-rise task performed at maximal voluntary effort: Peak rate of force development (RFD)	3D-magnetic resonance imaging (MRI): Muscle cross sectional area (CSA) of the right triceps surae, quadriceps and hamstrings muscle groups	Surface EMG: the rate of EMG rise for the right triceps surae muscles, soleus (SO) and medial gastrocnemius (MG)	*Faster vs slower* *Muscle performance* • No significance in usual 10-m walking speed between Faster and slower groups • Significant higher maximum 10-m walking speed and usual 400 m walking speed in Faster group • Plantarflexion RFD was lower in slower group *Muscle mass* • No significant difference in muscle CSA (triceps surae, quadriceps and hamstrings) between slower and faster group	*Faster vs slower* • Rate of EMG rise of MG was significantly lower in Slower Group • Rate of EMG rise of SO did not differ between groups *Correlation analysis* • Rate of EMG rise of MG was significantly positively associated with plantarflexion RFD, maximum walking speed and usual 400 m walking speed but not with usual 10-m walking speed	Maximum walking speed is limited by impaired neuromuscular force and activation of the triceps surae muscle group
**Clark et al. **[8]** (2013)**	Human	Male and female	75.0 ± 4.0 years	Quadriceps: Rectus femoris (RF) Vastus medialis (VM) Vastus lateralis (VL)	• Isometric maximal voluntary contraction (MVC) by one-repetition maximum (1-RM) method • Maximal speed leg press trials at a resistance setting equal to 70% of the 1RM (5 times): Leg press power	Computed tomography scans of the nondominant thigh	Surface EMG: rate of quadriceps EMG rise	*Baseline vs follow-up* *Muscle performance* • Significant reductions in leg press power in 3-year follow-up testing group • No significant difference in leg press one repetition maximum (1RM) strength between baseline and follow-up testing *Muscle mass* • Significant reduction in anterior thigh muscle cross-sectional area • No significant change in posterior thigh muscle cross-sectional area	*Baseline vs follow-up* • Significant reductions in the rate of EMG rise in follow-up testing group *Correlation analysis* • Loss of power was strongly associated with reduction in the rate of electromyogram rise, but not with reduction of anterior thigh muscle cross-sectional area	Voluntary neuromuscular activation declines with advancing age, contributes to a reduction in power production, and precedes the decline of mobility function
**Clark et al. **[9]** (2014)**	Human	Male and female	70–85 years	Rectus femoris (RF) Vastus medialis (VM) Vastus lateralis (VL)	• 400-m walk test • Isometric maximal voluntary contraction (MVC) against a resistance equal to 260 N (5 times)	Computed tomography (CT): Mid-thigh cross-sectional area (CSA), inter-muscular adipose (IMAT) and subcutaneous adipose tissues (SCAT)	Surface EMG to record peak EMG amplitude and neuromuscular activation quantified by rate of EMG rise	• The primary outcome measure of 400-m walking speed did not differ between males and females • In men, 400-m walking speed was strongly correlated with rate of EMG rise and moderately correlated with age, muscle CSA, and IMAT CSA • In females, 400-m walking speed was moderately correlated only with SCAT CSA	*Regression analysis* Rate of EMG rise of the quadriceps muscle group is a strong and independent predictor of walking speed in older males, but not in older females	Capability to rapidly activate the quadriceps muscle group is an important factor accounting for inter-individual variability of 400-m walking speed among older men, but not among older women
**Power et al. **[10]** (2016)**	Human	Male and female	82.8 ± 4.5 years 79.4 ± 3.7 years 79.3 ± 3.8 years 79.9 ± 6.2 years	Tibialis anterior (TA)	• Dorsiflexion maximal voluntary contractions (MVCs) (3 times)	N/A	• Surface EMG signals: compound muscle action potential (CMAP) amplitude, surface-detected motor unit potentials (S-MUP) amplitude and motor unit number estimates (MUNEs) • Intramuscular EMG: motor unit potential (MUP) size, near fiber (NF) area, duration, maximum NF interval, fiber count, NF jiggle, and NF jitter	*Male controls vs male masters athletes (MA) vs female controls vs female masters athletes (MA)* Significantly higher dorsiflexion strength in the MA groups (both males and females) compared with age-matched controls	*MA vs age-matched controls* • There were no interactions or main effects for sex among EMG parameters • Significant larger negative peak amplitude of the CMAP in MA group compared with age-matched controls • Decreasing trend of S-MUP in MA group compared with age-matched controls • Significantly larger MUNE in MA group compared with their age-matched counterparts • No significant difference in NF area, duration, and maximum NF interval values between MA and age-matched controls • Significantly smaller NF fiber counts, NF jiggle and NF jitter values in MA group *Correlation analysis* • Significant positive association between increasing S-MUP amplitude and increases in NF jiggle and NF jitter • Significant negative association with MUNE and NF jiggle and NF jitter	High-performing octogenarians better maintain neuromuscular stability of the MU and mitigate the loss of MUs associated with aging well into the later decades of life during which time the loss of muscle mass and strength becomes functionally relevant
**Piasecki et al. **[18] **(2016)**	Human	Male	26 ± 5 years 71 ± 4 years 69 ± 3 years	Tibialis anterior (TA)	• Maximal isometric voluntary contraction (MVC) of the ankle dorsiflexors • 25% maximum voluntary contraction force of the ankle dorsiflexors	• Magnetic resonance imaging (MRI): TA CSA • Dual-energy X-ray absorptiometry (DXA): Total lean mass, body fat percentage	• Surface EMG signals: CMAP amplitude, surface motor unit potential (sMUP) amplitude and MUNEs • Intramuscular EMG: motor unit potential (iMUP) area, firing rate, intramuscular MUNE, near fiber (NF) count, NF area, NF jiggle and NF MUP duration	*Young vs old vs master athletes* *Muscle performance* • Significant lower dorsiflexion MVC force in the old and master athletes than the young group *Muscle mass* • No significant difference in muscle CSA of the TA between young, old, and master athlete groups • Significant higher body fat percentage in the old compared with young • Significant lower body fat percentage in the master athletes compared with old • Significantly lower total weight in the master athletes compared with young • Significantly lower total lean mass in old and mater athletes than the young	*Young vs old vs master athletes* *Surface EMG* • Significantly higher CMAP of the TA in the young compared to old and master athletes but there was no significant difference between old and master athletes • Surface MUP amplitude did not differ between the three groups *Intramuscular EMG* • Significantly higher iMUP areas in the old and master athletes compared with the young, with no significant differences between old and master athletes • Significantly lower mean firing rate in the old and masters athletes compared to the young, with no difference between the old and master athletes • Significantly higher NF Jiggle in the Master Athletes compared to the Young, with no difference between Master Athletes and Old • No significant difference in NF Count between the three groups • Significant higher NF Area and Duration in the Old and Master Athletes than the Young, with no difference between the older groups • Significantly lower iMUNE in Old and the Master Athletes than the Young, with no difference between Old and Master Athletes	Substantial and similar motor unit loss and remodeling in master athletes and old individuals compared with young, which suggests that lifelong training does not attenuate the age-related loss of motor units
**Gilmore et al. **[20] **(2017)**	Human	Male and female	75.3 ± 6.5 years 77.9 ± 7.0 years 82.0 ± 4.7 years	Tibialis anterior (TA)	• Handgrip strength • Gait speed • Dorsiflexion maximal voluntary contractions (MVCs): strength, maximal MVC rate of torque development (RTD), normalized MVC RTD • Voluntary activation capacity of the dorsiflexors by interpolated twitch technique (ITT): Voluntary activation percentage, peak twitch tension, time to peak tension (TPT), normalized twitch RTD, half-relaxation time (HRT), negative peak relaxation rate	Ultrasound imaging: muscle thickness (MT) of the tibialis anterior (TA)	• Surface EMG signals: CMAP amplitude, sMUP amplitude and calculate MUNEs • Intramuscular EMG: motor unit potential (MUP) duration, contractions, motor unit potential trains (MUPTs), near fiber (NF) area, NF duration, NF dispersion, maximum NF interval, NF fiber count, NF jiggle and NF jitter, blocking	*Pre-sarcopenia vs sarcopenic vs severe sarcopenic* *Muscle mass* • No significant differences among the 3 sarcopenia stages were found for tibialis anterior MT *Muscle performance* • No significant differences in voluntary activation percentage, absolute/normalized TPT and half-relaxation time and negative peak relaxation rate between 3 groups • Significantly lower MVC in the severe sarcopenia group compared with the sarcopenic and pre-sarcopenic groups • Significantly lower Peak twitch tension in the severe sarcopenia group compared with pre-sarcopenic group	*Pre-sarcopenia vs sarcopenic vs severe sarcopenic* *Surface EMG* • No statistically significant differences for CMAPs, mean SMUPs and MUNEs among the 3 sarcopenia groups *Intramuscular EMG* • MUP duration, contractions, MUPTs/contraction, NF area, NF duration, NF dispersion, maximum NF interval were not significantly different among 3 groups • Significant higher NF jiggle in the severe sarcopenia group compared with the pre-sarcopenia group and sarcopenia group • Significant higher NF jitter in the severe sarcopenia group compared with the pre-sarcopenia group, and a trend toward a difference between the sarcopenia and severe sarcopenia groups • Significantly higher NF jitter in the sarcopenia group compared with the pre-sarcopenia group • None of the participants in any category had MUPT blocking *Correlation analysis* • NF jiggle was positively correlated with mean SMUP amplitude • Negative relationships between jiggle and MUNE	• All sarcopenia groups underwent MU remodeling, but that the severe sarcopenia group was more compromised in this process than the pre-sarcopenia group • Pre-sarcopenia is reflected by the higher quality of collateral reinnervation exhibited by lower jiggle and jitter values when compared with severe sarcopenia
**Olmos et al. **[12]** (2019)**	Human	Male	45.1 ± 2.7 years 65.3 ± 3.2 years	Gastrocnemius	• Handgrip strength • Concentric, isotonic and isokinetic plantar flexors (PF) contractions: isometric peak torque (PTI_SOM_), dynamic peak torque (PT_DYN_), peak power (PP), intensity, specific PP, rate of torque development (RTD), rate of velocity development (RVD), rate of power development (RVD), optimal torque (OPT_T_) and (optimal velocity (OPT_V_)	• Dual-energy X-ray absorptiometry (DXA): Body fat percentage • Ultrasound: CSA of medial and lateral MG, echo intensity (EI), muscle quality and subcutaneous fat thickness	• Surface EMG signals to record rate of rise in electromyography rise (RER) during the peak power contraction • ELISA: serum concentrations of C-terminal agrin fragment (CAF) as indicative of NMJ deterioration	*Middle-aged vs older* • All characteristics were similar between groups: body fat%, appendicular lean mass, EI, PT_DYN_, PTI_SOM_, specific PT_DYN_, PP intensity, specific PP, OPTV, OPTT, and RVD • A trend of difference in handgrip strength and PTI_SOM_ between groups • PP, RPD, and RTD were significantly lower in older males compared with middle-aged males *Correlation analysis* • RTD was the sole predictor of peak power	• RER were significantly lower in older males compared with middle-aged males • CAF levels were similar between groups, and not associated with RER, PP, or time-dependent contractile parameters	Middle-aged and older males produced peak power at the same relative intensity and velocity, but neuromuscular functioning and torque capacity, specifically RTD, was lower in older males
**Sawers et al. **[13]** (2017)**	Human	Female	≥ 65 years	Tibialis anterior (TA) Medial gastrocnemius (MGAS) Vastus lateralis Biceps femoris long head (BFLH)	Laboratory-induced slip and fall trial: 7-m walkway at a self-selected speed • Slip-related variables: peak slip velocity, slip duration, slip distance • Gait-related variables at slip onset: Shank angle, dynamic stability, walking speed	N/A	Surface EMG activity: onset latencies and peak activity	*Fall vs recover* • No statistically significant difference in slip duration or peak slip velocity among subjects who fell compared with those who recovered during slip trials • Significantly greater slip distance in fall group compared with recovery group • No statistically significant difference between the fall and recovery groups in whole body stability, walking speed or shank angle at slip onset	*Fall vs recover* • Muscle onset latency was longer among subjects who fell compared with those who recovered during slip trials • Significantly longer onset latencies for BFLH and VLAT muscle in the leading/slip leg in fall group compared with recover group • No differences were found in peak muscle activity between groups	Falls may arise from differences in neuromuscular control of walking and balance that are characterized by lower complexity of neuromuscular control during slip and nonslip trials, delayed temporal coordination of muscle activity about the knee and excessive coactivation during slip responses, as well as greater interlimb muscle coordination during slip and nonslip conditions
**Soderberg et al. **[14]** (1991)**	Human	N/A	23–35 years (mean 31) 64–82 years (mean 76)	Abductor digiti minimi	• 10% maximum voluntary contraction: Force monitored via Statham UC3 transducer • 10-s ramped protocol (5s up, 5 s down): Force trajectory: Linear ramp up and down • Recording speed: 15 in./s	N/A	Surface EMG: interspike interval calculation • Visual identification of single motor unit • action potentials (SMUAPs) • ISI = time between successive SMUAPs	N/A	Interspike interval differences • Young subjects: 88.4 ms (concentric), 96.5 ms (eccentric) • Aged subjects: 110.5 ms (concentric), 117.1 ms (eccentric) • 25% longer firing intervals in elderly • Consistent pattern across both contraction types Firing pattern variability • Greater standard deviation in aged subjects • Concentric: 69.9 ms (aged) vs 52.3 ms (young) • Eccentric: 60.4 ms (aged) vs 52.8 ms (young) • Indicates less stable motor unit control	• Elderly subjects showed difficulty modulating firing rates during lengthening contractions • Less adaptive response to changing force requirements • May explain clinical observations of movement quality differences
**Akataki et al. **[27]** (2002)**	Human	Male	22.7 ± 1.8 years 69.8 ± 4.7 years	Biceps brachii	• Maximum voluntary contraction: force • Ramp contractions: 10% MVC for 3 s → linear progression 10–80% MVC	N/A	Mechanomyogram (MMG) recording system: root mean squared amplitude (RMS), Mean power frequency (MPF) of the mechanomyogram (MMG) relative force (% MVC)	• Elderly MVC: 41.3 ± 8.4 Nm • Young MVC: 59.1 ± 6.8 Nm	Motor unit changes with aging • Preferential loss of fast-twitch motor units (FT − MU) • Increased reliance on slow-twitch motor units • Reduced total motor unit number • Increased innervation ratio per remaining unit Firing rate adaptations • Lower firing rates in elderly • Effective fused tetanus at reduced frequencies • Prolonged contraction duration enables fusion at lower rates Recruitment strategy alterations • Extended ST-MU recruitment phase • Delayed FT-MU recruitment • Reduced mechanical power from remaining FT-Mus • Compensatory mechanisms for force production • RMS amplitude increased progressively up to 57.6% MVC, then brief rapid increase followed by stable trend beyond 63.6% MVC • Mean power frequency MPF increased slowly up to 59.4% MVC, temporary reduction to 64.3% MVC, then progressive increased	• Elderly showed markedly different MMG patterns compared to young adults • Both RMS and MPF were smaller in elderly throughout submaximal force levels • Results reflect altered MU activation strategy with predominant role of slow-twitch fibers and effective • Fused tetanus at lower firing rates
**Galea et al. **[15]**. (1996)**	Human	Male and Female	20–98 years	Thenar, Bicepsbrachii, Extensor digitorum brevis (EDB)	N/A	N/A	Surface EMG: motor unit estimation system • M-wave (compound action potential) measurements as an indicator of excitable muscle fiber mass: peak-to-peak amplitude, voltage–time integral (area) • Motor unite action potential (MUAP): amplitude, duration, configuration, area	M-wave changes (muscle fiber mass): • Peak-to-peak amplitude reduced in all muscles • EDB and biceps showed greatest reductions (~ 50% in > 80 years) • M-wave area changes prominent in EDB, less severe in biceps/thenar	Proximal muscle (biceps) • No significant change in motor unit numbers with age • Motor unit estimates remained within young adult range • MUAP/M-wave ratio remained constant Motor unit loss: • Significant loss only after age 60 years • Distal muscles more susceptible than proximal	• Motor unit populations decreased significantly with age in distal muscles thenar and EDB • After age 60, but remained constant in the proximal biceps muscle • Excitable muscle fiber mass M wave amplitude and area was diminished in all three muscles • The ratio of average motor unit action potential to M-wave area increased with age in distal muscles, suggesting collateral reinnervation
**Willoughby et al. **[16]** (2024)**	Human	Female	Pre-menopausal (PRE-M) post-menopausal (POST-M)	Vastus lateralis Rectus femoris	• One repetition maximum (1RM) for upper- and lower-body strength • Repetitions to failure (RTF) at 70% of the leg press and chest press 1RM for the assessment of lower- and upper-body muscle endurance • Muscle quality (MQ): upper-body MQ—upper-body relative strength divided by the sum of right and left arm fat free mass (FFM) and trunk FFM; lower-body MG—lower-body relative strength values divided by the sum of left leg FFM and right leg FFM	• Dual-energy X-ray: percent body fat (PBF), fat mass (FM), fat-free mass (FFM), and visceral fat (VFM) • Standard scale: total weight	Surface EMG for motor unite function • Maximum EMG activity during RTF • Mean EMG activation during RTF Neuromuscular junction: • Enzyme-linked immunosorbent assay (ELISA) kits for blood samples: neurofilament light chain (NFL), C-terminal agrin fragment (CAF), neurotrypsin (NT)	*Muscle mass* • Increased total body mass, percent body fat, and visceral fat in POST-M • No significant differences in fat-free mass between groups • Typical age-related body composition shifts *Muscle performance* • Upper body muscle quality: POST-M 0.02 vs PRE-M 0.05 RS/FFM • Lower body muscle quality: POST-M 0.09 vs PRE-M 0.13 • Reduced relative strength despite similar fat-free mass RS/FFM *Muscle proteolysis* • POST-M women showed significantly decreased estradiol E2 26.36 vs 399.41 pg/mL levels • Urinary titin N-terminal fragment: POST-M 20.69 vs PRE-M 11.1 ng/mL	Motor unit function • Significantly reduced motor unit activation in POST-M women • Decreased maximum EMG activity in vastus lateralis and rectus femoris • Impaired neuromuscular recruitment patterns Neuromuscular junction degradation: CAF: POST-M 3860.20 vs PRE-M 1208.40 pg/mL	Early to intermediate post-menopausal women experience a cascade of physiological changes beginning with decreased estradiol, leading to increased inflammation, neuromuscular junction degradation, and reduced muscle function—potentially exacerbated by inadequate protein intake
**Soga et al. **[30]** (2015)**	Human	Male	31–48 years	Vastus medialis	Maximal isokinetic extension strength of each leg	Bioimpedance body fat analyzer: body weight, body fat ratio, and muscle mass	Surface electromyography The root mean square (RMS) amplitude	Muscle mass No overall changes in body weight, body fat ratio, whole-body and leg muscle mass, or thigh circumference were observed in milk fat globule membrane (MFGM) and placebo groups after 4 weeks exercise Muscle performance • MFGM group: + 4.23% increase from baseline • Placebo group: − 0.95% decrease from baseline	Electromyography results MFGM group: + 12.52% increase in RMS amplitude Placebo group: − 8.92% decrease in RMS amplitude	MFGM supplementation enhances exercise-induced muscle strength improvements through neuromuscular mechanisms, potentially offering an efficient strategy for maintaining muscle function in middle-aged adults
**Kent-Braun et al. **[28]** (2002)**	Human	Male female	Young women (*n* = 10): 32.3 ± 4.8 years Young men (n = 10): 33.5 ± 6.5 years Older women 75.0 ± 5.9 years Older men 74.4 ± 5.3 years	Dorsiflexor muscles	Muscle force • MVC force (N) • Tetanic force (50-Hz, 500-ms stimulation) • Twitch force (0.1-ms pulse) • Potentiated twitch force Contractile Properties • Maximum rate of force development (dF/dt) • Maximum rate of force relaxation (dF/dt) • Twitch contraction time (ms) • Tetanic half-relaxation time (ms) • Time to peak force	N/A	Surface electromyography: Activation measures • Central: Central Activation Ratio (CAR = MVC/(MVC + superimposed force)) • Peripheral: Compound muscle action potential (CMAP) amplitude and duration • Neural drive: Motor unit discharge rates and recruitment patterns	Age effects on fatigue • Older subjects fatigued less than young subjects • Postexercise MVC/pre-exercise MVC: older adults showed greater fatigue resistance • No age-related differences in central or peripheral activation failure Gender effects on fatigue • No significant effect of gender on overall fatigue • Gender differences observed in metabolic responses rather than fatigue outcomes Strength-fatigue relationship • Modest correlation between pre-exercise strength and fatigue • 25% of fatigue variability related to muscle strength • Stronger subjects showed greater fatigue, possibly due to higher intramuscular pressure	Central activation • No age or gender differences in central activation ratio CAR • No central activation • failure in any group during this moderate fatigue protocol • Contradicts some previous reports of age-related central activation impairment Peripheral activation • CMAP amplitude lower in older vs. young subjects at baseline • No peripheral • activation failure during exercise in any group • Similar potentiation responses across age groups	• Older adults may have metabolic advantages during submaximal exercise • Dorsiflexor muscles are functionally important for locomotion, posture, and fall prevention • Results may inform exercise prescription and rehabilitation strategies for different age groups and genders
**Sarto et al. **[21]** (2024)**	Human	Male Female	25.98 ± 4.6 years 75.9 ± 4.7 years	Quadriceps Vastus lateralis	• Maximum voluntary contraction (MVC) • Grip strength • Rapid force production	• DXA: Appendicular lean mass (ALM) for sarcopenia classification: non-sarcopenic (NS), pre-sarcopenic (PS), or sarcopenic (S) • Panoramic ultrasound imaging: quadriceps femoris cross-sectional area (CSA) • Muscle architecture: fascicle length (Lf) and pennation angle (PA)	• Intramuscular electromyography (iEMG): motor unit potential (MUP) area, duration, number of turns, mean firing rate • Near fiber MUP: duration, NF count/fiber density contributing, segment jitter • Motor unit number estimate (iMUNE) = mean MUP area/vastus lateralis CSAmean	*Muscle mass* Appendicular lean mass (ALM) • Significant decrease across sarcopenia stages • Progressive reduction from NS → PS → S groups • ALM/height^2^ also showed diminishing trend Quadriceps cross-sectional area • Gradual decrease across sarcopenia stages • Mean CSA at 30%, 50%, and 70% of femur length all reduced Vastus lateralis • CSA mean significantly reduced • Fascicle length: No differences among groups • Pennation angle: lower in all older groups vs. young Leg lean mass • Significant reduction across stages • Consistent with overall pattern of muscle atrophy • Progressive loss from non-sarcopenic to sarcopenic *Muscle function* • Handgrip strength increasingly lower from NS to S • Knee extensor maximum voluntary contraction MVC significant reduction across stages • Slower capacity in older groups of rapid force production, no differences between NS, PS, and S groups	Motor unit loss • iMUNE: Lower in all older groups vs. young • No difference between NS, PS, and S groups NMJ transmission impairment • NF segment jitter: Elevated in all older groups at 25% MVC • Y vs. NS/S: *p* < 0.0001; Y vs. PS: *p* = 0.0169 Motor unit complexity changes • MUP turns: elevated in NS vs. Y at 25% MVC (*p* = 0.0278) • -NF MUP duration: Increased in older groups (*p* = 0.003) • NF count: higher in NS and PS vs. Y at both intensities Firing rate changes: Reduced in Sarcopenic: S vs. Y at 25% MVC (*p* = 0.039)	• Gradual decrease in muscle force, cross-sectional area, and lean mass across sarcopenia stages • Elevated near fiber segment jitter in all older groups, indicating impaired NMJ transmission • Increased C-terminal agrin fragment and altered caveolin 3 expression suggesting NMJ instability • Lower motor unit number estimate iMUNE • In all older groups, confirming age-related MU loss • Increased muscle denervation and axonal damage markers • Importantly: most neuromuscular parameters did not differ between older individuals with or without sarcopenia
**Stålberg et al** [85]** (1975)**	Human	N/A	10–89 years	Digitorum communis	N/A	N/A	Single-fiber electromyography (SFEMG): Jitter, blocking detection, duration measurement, multiple potential analysis	N/A	Age-related fiber density changes • Ages 20–69: gradual increase (1.38–1.55) • Ages 70–79: accelerated increase (1.78) • Ages 80–89: marked increase (2.32) • Ages 10–19: unexpectedly high values (1.50) Spike component distribution • Single potentials: ~ 60–65% across most ages • Double potentials: ~ 30–35% • Triple potentials: ~ 5% • Multiple components increase after age 70	• Fiber density increases slowly throughout life with accelerated progression after age 70 • Increased impaired nerve transmission with aging • Evidence of degenerative motor neuron loss compensated by reinnervation • EDC muscle shows relatively slight changes compared to other muscles, making it suitable for detecting early pathological changes independent of age factors
**Shima et al. **[26]** (2007)**	Human	Male	27.1 ± 3.8 years 79.0 ± 2.5 years	Dorsiflexor muscles	• Supramaximal electrical stimulation • 10-s maximal voluntary contraction • Post-activation potentiation assessment • Submaximal contractions (20%, 40%, 60%, 80%, 100% MVC)	N/A	• Mechanomyographic (MMG): Baseline to initial positive peak (evoked) • EMG: peak-to-peak M-wave amplitude • RMS calculation of the voluntary EMG and MMG signals	N/A	Electrically evoked responses • Peak twitch torque potentiation greater in young vs old • MMG amplitude potentiation similar between groups • M-wave amplitude unchanged in both groups • Duration: torque potentiation lasted ≥ 5 min, MMG only 2–3 min Voluntary contraction responses • EMG-torque relationships similar between age groups • MMG-torque: linear increase in young, blunted response in old • Young had significantly greater MMG at 100% MVC • Different curve shapes between MMG and EMG in elderly	• Twitch potentiation was greater in young than old subjects • Evoked MMG potentiation was similar between age groups • Voluntary MMG-torque relationships differed: young showed linear increase, old showed blunted response • EMG-torque relationships were similar between groups • Young men had greater MMG at maximum voluntary contraction
**Motanova et al. **[29]** (2025)**	Human	Male	68.5 ± 2.6 years	Vastus lateralis	N/A	N/A	• Intramuscular electromyography (iEMG): motor unit potential (MUP) area, duration, number of turns, mean firing rate, • Near fiber MUP: Duration, NF count/fiber density contributing, segment jitter	N/A	Motor unit properties: • Firing Rate: Decreased at both 25% and 50% MVC intensities • MUP Complexity: Increased number of turns and temporal dispersion • Area changes: reduced MUP area reflecting smaller motor unit size NMJ transmission impairment: Increased jiggle & jitter: higher near-fiber jiggle and segment jitter indicating compromised transmission	This is the first study to directly demonstrate human NMJ morphological changes induced by inactivity, suggesting older adults are particularly vulnerable to neuromuscular dysfunction during periods of disuse
**Balci et al. **[24]** (2005)**	Human	Male Female	73.9 ± 1.7 years 82.2 ± 1.2 years	Frontalis	N/A	N/A	Single-fiber electromyography (SFEMG): Jitter		• Normal jitter: 40.4 μs 70–79 years; 43.7 μs > 80 years • Normal fiber density limits: 1.90 70–79 years; 2.14 > 80 years	Reference values may have value to diagnose neuromuscular transmission abnormalities in elderly patients
**Guo et al. **[23]** (2025)**	Human	Male Female	60–83 years	Vastus lateralis Tibialis anterior	Grip strength Timed up and go (TUG) Maximal voluntary contraction Force steadiness assessment • Target line at normalized contraction intensity • Coefficient of variation (CoV) calculation: (SD/mean) × 100 • Measured at 10% and 25% MVC levels	Ultrasound imaging: cross-sectional area (CSA) measurement of vastus lateralis	• Motor unit firing rate (MUFR) • Motor unit potential (MUP): Area, phases: • Near fiber motor unit potential jiggle • Intramuscular electromyography (iEMG)	*Muscle mass* • Females had 39.3% smaller vastus lateralis CSA compared to males • Age-related decline: 0.29 cm^2^ decrease per year *Muscle function* • Knee extensor torque: females: 46.3% lower than males; age decline: 3.10 Nm per year • Grip strength: females: 43.1% lower than males; age decline: 0.43 kg per year • Force control and steadiness: Females showed poorer force steadiness at both contraction levels: 21.3% worse at 10% MVC; 14.5% worse at 25% MVC; age-related decline in force steadiness from early to late elderly • Force steadiness progressively decreased at 10% MVC • Timed up and go (TUG): females—10.1% longer completion time	• Motor unit number: ~ 30% fewer motor units in quadriceps and tibialis anterior of older vs younger males • Motor unit firing rate: Females show consistently higher firing rates, possibly compensating for remodeling effects* • No Sex Differences in MUP Duration, area, complexity, NFM Jiggle	• Older females showed higher motor unit firing rates than males at normalized contraction levels, consistent with patterns seen in young adults • Females had significantly smaller muscle cross-sectional area, lower strength, and poorer force control • Both sexes showed similar trajectories of neuromuscular deterioration from early to late elderly, but males consistently maintained superior function

**Table 2:** Characteristics of Preclinical studies

Study	Species	Gender	Age	Intervention	Muscle	Muscle strength measurements	Muscle mass measurements	NMJ morphology	Neurophysiological measurements
**Fahim et al. **[49]** (1997)**	C57BL/6NNia mice	Male	7 or 25 months	Rodent treadmill: 28 m/min for 60 min/day, 5 days/week for 12 week	Gluteus maximus	Nerve-evoked isometric twitch tension in standard Krebs solution and reduced-Ca^2+^ solution	• Body weight • Muscle fiber diameter	Nerve terminal stained by zinc iodide-osmium (ZIO) technique: nerve terminal perimeter, nerve terminal area, integrated area within the nerve terminal, longitudinal extent length, number of nerve terminal branches	Intracellular EMG recordings: resting membrane potential (*E*_m_), miniature endplate potentials (MEPP) amplitude, MEPP rise time, MEPP ½ decay time, MEPP frequency, muscle fiber input resistance of electrodes (*R*_in_) and membrane capacitance, endplate potentials (EPP) amplitude, calculated quantal content
**Chung** **et al. **[37]**(2017)**	C57BL/6J mice	Male	2–4 months old 22–25 months old	N/A	Gastrocnemius soleus	Grip strength of forearm and four limbs	N/A	• Immunofluorescent quantification of NMJs: postsynaptic motor endplates morphology, muscle fiber denervation, endplate area and AChR occupancy • Lumbar ventral root nerve morphometry: total axon counts per root, axon counts per area, axon diameter, or G-ratio (ratio of axon diamater to fiber diameter)	Stimulated single-fiber electromyography: jitter
**Iyer** **et al. **[46]**(2021)**	C57BL/6J mice	Male	12 months old 24 months old 27 months old	Follistatin (FST) overexpression delivered via adeno-associated virus subtype 9 injection	Gastrocnemius Tibialis anterior Triceps brachii	Longitudinal twitch and tetanic plantar flexion contractility testing: peak twitch torque and tetanic torque	Muscle weights	Immunohistochemistry of NMJs: innervation junctions and NMJ fragmentation	• Needle EMG to record compound motor action potential (CMAP) amplitude, single motor unit potential (SMUP) amplitude and MUNE • Single-fiber electromyography (SFEMG) to record NMJ jitter and blocking
**Chung et al. **[43]** (2018)**	C57BL/6J mice	Male	3–5 months old 20–24 months old	N/A	Gastrocnemius	• Grip strength of forelimbs and all four limbs • Behavioral evaluation: traveling and standing activity • Treadmill test: number of shocks (NOS)	Body weight	N/A	• Needle EMG to record compound motor action potential (CMAP) amplitude, single motor unit potential (SMUP) amplitude and MUNE • Single-fiber electromyography (SFEMG) to record NMJ jitter
**Chugh et al. **[44]**(2020)**	C57BL/6J mice	Male and female	12, 20, 24, 27, 29 months old	N/A	Tibial innervated plantar flexor muscles Gastrocnemius and soleus Tibialis anterior	• Frailty index: evaluation of the integument (fur loss, coat color, coat condition, and dermatitis), musculoskeletal system, vestibulocochlear/auditory system, ocular and nasal systems, digestive system, urogenital system, respiratory system, signs of discomfort, body weight, and body surface temperature • In vivo muscle contractility: maximum torque during a train of stimulation at 150 Hz	Body weight	N/A	• Single-fiber electromyography (SFEMG): single muscle fiber action potential, jitter, and blocking • Frequency dependency SFEMG: single muscle fiber action potential jitter and blocking • Ex vivo neuromuscular junction electrophysiology: miniature endplate potential current (MEPC) and frequency, endplate current, and quantal content
**Padilla et al. **[34] **(2021)**	F344 rats	Male and female	6 months old 26 months old	N/A	Gastrocnemius Forelimb flexor	Bilateral forelimb and hindlimb grip strength In vivo plantar flexion muscle contractility: peak absolute maximal torque values, peak torque values normalized to body mass and muscle mass and the % decline of plantar flexion torque	Muscle weight	N/A	• Repetitive nerve stimulation (RNS) of 10 supramaximal stimuli delivered to the sciatic nerve at 10, 20, 30, 40, and 50 Hz: to record repetitive CMAP amplitudes • Stimulated single-fiber electromyography (SFEMG) to record NMJ jitter and blocking
**Ham et al. **[39]** (2020)**	C57BL/6JRj mice	Male	8 to 30 months old	Rapamycin	Gastrocnemius (Gas) Tibialis anterior (TA) Extensor digitorum longus (EDL) Quadriceps Plantaris Soleus	• Voluntary running-wheel analysis: the total distance run in the 24-h period • Grip strength of forarm and all limb • Gait analysis: stride length, wing speed and duration, duty cycle, base of support and stand time • Comprehensive laboratory animal monitoring system (CLAMS): locomotor activity, energy expenditure, oxygen consumption (VO_2_), CO_2_ production (VCO_2_), and the respiratory exchange ratio (RER) • In vitro muscle force of EDL and soleus and nerve versus muscle-stimulated force of soleus	• Body composition analyzed using EchoMRI-100: fat and lean mass • Immunostaining of muscle cross sections: muscle fiber typing	Whole-mount NMJ staining: number of axonal inputs, NMJ terminal sprouting, and AChR fragments	• In situ electrophysiological recordings of single TA and soleus NMJ: miniature end-plate current (mEPC), end-plate current (EPC), quantal content, and EPC over repeated stimulations • Repetitive nerve stimulation electromyography of gas: CMAP
**Pannerec et al. **[36]** (2016)**	Wistar rats	Male	Adult: 8–10 months Early-sarcopenic: 18–20 months Sarcopenic: 22–24 months	N/A	Gastrocnemius, tibialis anterior (TA), extensor digitorum longus (EDL) Biceps brachii, triceps brachii	• Functional assessment via gait analysis using the Catwalk XT system (Noldus): gait speed, stride length, stand time, and swing time were measured. Animals walked on a glass lane with camera-based paw print detection	• Direct measurement of muscle weight after dissection, normalized to body weight • Muscle fiber cross-sectional area assessed by immunofluorescence histology	Fluorescently conjugated α-bungarotoxin (labels acetylcholine receptors): Each NMJ was classified into three classes based on number of fragments (1–2, 3–4, ≥ 5 fragments) counted manually; about 50 NMJs per muscle analyzed	• Needle electromyography (EMG): Compound muscle action potential (CMAP) was recorded by stimulating nerves (sciatic for hindlimb, radial for forelimb • Motoneuron retrotracing: Fluorogold was injected into target muscles; after 10 days, labeled motoneurons in the spinal cord were counted via fluorescence microscopy of spinal cord sections
**Banerjee et al. **[60]** (2024)**	Drosophila melanogaster (fruit fly)	Male	Days 3, 24, 40	Trio knockdown/overexpression, UAS-Trio, and UAS-human Trio transgenes expressed in adult motor neurons GEF domain mutants Miniature neurotransmission manipulation	Abdominal A2 musculi ventralis interni mediales (MVIM)	Negative geotaxis (climbing) assay	N/A	• Immunohistochemistry and confocal imaging • Confocal microscopy • STED super-resolution microscopy	Electrophysiology: • Excitatory junctional current (EJC) amplitude: standard single-pulse evoked release • Miniature EJC (mEJC) amplitude and frequency: spontaneous neurotransmitter release events • High-frequency stimulation: high-intensity neurotransmitter release (mean EJC amplitude averaged every 10 s) • Readily releasable pool (RRP) measurements were also performed Rac activity at synapses: • Rac-FRET biosensor (Raichu-Rac)
**Azpurua et al. **[61]** (2018)**	Drosophila melanogaster (fruit fly)	Not specified	Days 7, 35, 45 Addtional time points Days 14, 21, 37	Overexpression of dMMP1 in motor neurons Overexpression of human TDP-43 and mutant forms (ALS models) Dietary restriction (19% vs. 29% yeast diets)	Cibarial muscle 9 (CM9)	• Negative geotaxis (“wall climbing”) assay: Measures the ability of flies to climb an 8-cm threshold in a vertical vial within 10 s after being tapped down. The percentage of flies crossing the threshold was recorded	N/A	• Dissections of CM9 muscle were stained with antibodies to synaptic vesicle protein VGluT (presynaptic marker) and discs-large (DLG, the postsynaptic PSD-95 homologue) • Confocal microscopy: the number of boutons per muscle fiber and total synaptic volume (measured by masking total VGluT signal from boutons) • 3D reconstruction of NMJs was performed to localize dMMP1 at the synapse	• Electrophysiological recordings at the CM9 NMJ in adult flies • Measured evoked excitatory postsynaptic potentials (EPSPs) and miniature EPSPs (mEPSPs) • Calculated quantal content (QC) as a measure of neurotransmitter release
**Sugita et al. **[58]** (2021)**	BALB/c and C57BL/6J mice	Male	Experiment 1: 6 to 23 months Experiment 2: 18 to 24 months 6 months old (BALB/c), 10 months old (C57BL/6J)	Control diet (CONT/Cont) MFGM diet (MFGM) Control diet with exercise (EX-CONT/Ex-Cont) MFGM diet with exercise (EX-MFGM/Ex-MFGM)	Extensor digitorum longus (EDL) Soleus Tibialis anterior (TA) Gastrocnemius EDL	• Rotarod test: 4 rpm to 40 rpm over 5 min	Muscle dissection and weighing	• NMJ immunostaining: Fragmented AChRs (≥ 5 clusters), denervated NMJs • Peroneal nerves: Axon numbers, myelin abnormalities (inclusions, outfoldings, tomacula), and g-ratio (axon/nerve fiber diameter) • Neurofilament immunostaining for axon • Spinal cords staining for Motor neuron and caspase-3-positive percentages	• Motor nerve conduction velocity (MNCV): M-waves and velocity
**Kelly** **et al. **[51] **(1986)**	CBF-1 mice (CB6F, BALB/cNNia × c57BL/6NNia)	Male	8–9 months 28–30 months	N/A	Soleus muscle Extensor digitorum longus (EDL)	N/A	Weighing dissected muscle regions	N/A	Electrophysiological recordings: End-plate potentials (epp’s) and resting membrane potentials (rmp’s), quantum content
**Kelly** **et al. **[52] **(1986)**	Mouse (CBF-1)	Male	12 months 30 months	N/A	Soleus Diaphragm	N/A	N/A	N/A	Intracellular recording: spontaneous miniature end-plate potentials (mepps) and evoked end-plate potentials (epps): amplitude, frequency, rise time, and half-decay time
**Zhang et al. **[40]** (2024)**	C57BL/6J mice	Male and female	0.5, 1.5, 2, 4, 5, 6, 7, 18, and 24 months	Genetic manipulation: Skeletal muscle-specific Sirt6 knockout (HSA-Sirt6 cKO) Pharmacological intervention: NMN (β-Nicotinamide mononucleotide): Orally administered at 1.5 mg/ml in water to 18-month-old mice daily for 6 months	Tibialis anterior (TA) Gastrocnemius Diaphragm Hemidiaphragm	Grip strength Rotarod test: latency to fall	N/A	• Whole-mount staining of muscles: NMJ fragmentation, size, and morphology	• Needle electromyography (EMG): compound muscle action potential (CMAP) • Electrophysiological recording: miniature end plate potentials (mEPPs)
**Zhao et al** [41]** (2018)**	C57BL/6 mice	Male and female	3, 24 months	Transgenic expression: Generation of Flag-Lrp4 transgenic mice with muscle-specific expression under the human α-skeletal actin (HSA) promoter AAV9-mediated gene delivery: AAV9 vectors expressing human SGCA (SGα) or GFP	Tibialis anterior (TA) Gastrocnemius Diaphragm	In vivo twitch and tetanic force measurement: Tetanic contractions	Muscle dissection and weighing	• Whole-mount immunofluorescence: Fragmentation, innervation, AChR cluster intensity	• Needle electromyography (EMG): compound muscle action potentials (CMAPs) • Electrophysiological Recording: Miniature End Plate Potentials (mEPPs)—Frequency and amplitude
**Kelly** **et al. **[50] **(1983)**	CBF-1 strain mice (BALB/cNNia × C57BL/6NNia)	Male	7–8, 25 months	N/A	Extensor digitorum longus Soleus Diaphragm	The indirect twitch response: The nerve was stimulated via a suction electrode with twice maximal stimulus strength	N/A	N/A	Electrophysiological recording: Miniature End Plate Potentials (mEPPs)—Frequency and amplitude and quantal content
**Hu et al. **[59]** (2023)**	Worms: *Caenorhabditis elegans* (*C. elegans*) C57BL/6 mice	Worms: Not gender-specified Mice: Male	Worms: D1, D3, D5, D7, D9, D11, D13, D15, D17 Mice: 16 months	Genetic manipulations in worms: Vps-34(qx546) mutant worms Transgenic overexpression or rescue lines Pharmacological intervention: SAR405 (VPS34 inhibitor) Mice: 5 mg/kg SAR405, daily intraperitoneal injection for 1 month	Worms: Body wall muscles (for locomotion and mitochondrial integrity studies) Mice: Tibialis anterior (TA)	Worms: • Motor behavior assays: Body bends, thrashing: • Treadmill-like “out-of-circle” assay Mice: • Treadmill running test	N/A	Worms: • Synaptic puncta quantification: • GFP-tagged synaptic vesicle proteins (GFP:RAB-3, SNB-1:GFP) under neuron-specific promoters imaged in dorsal nerve cord; puncta number quantified at D3 and D11 for excitatory (unc-129) and inhibitory (unc-25) neurons • No specific morphological NMJ analysis in mice	Worms: • Electrophysiology (NMJ function): In situ whole-cell patch-clamp recordings of anterior muscle cells at the ventral nerve cord NMJ: endogenous miniature postsynaptic currents (mPSC), frequency and amplitude
**Zitman et al. **[86]** (2011)**	Mice: GM2/GD2-synthase knockout (GM2s-KO), GD3-synthase knockout (GD3s-KO), and wild-type (WT) littermates	Male and female	9.5–17 months	Genetic interventions: GM2s-KO mice, GD3s-KO mice	Diaphragm	Grip strength	Body weight	N/A	In vitro electrophysiology on Diaphragm-phrenic nerve: Intracellular recordings of miniature endplate potentials (MEPPs) and endplate potentials (EPPs) at NMJs, quantal content
**Miyamaru n et al** [47]** (2012)**	Wistar rats	Female	2, 20 months	Denervation-only (DNV): RLN transection without further intervention Nerve-muscle pedicle (NMP) implantation: RLN transection immediately followed by NMP implantation	Thyroarytenoid muscle of the larynx	N/A	N/A	Immunohistochemistry was used to assess neuromuscular junctions (NMJs): number of NTs and AChRs were counted, and ratios (T/U) were calculated for each side	Evoked EMG: Muscle action potentials (MAPs)—The amplitude of MAPs in the TA muscle was measured on both sides after supramaximal nerve stimulation. The ratio of treated/untreated side (T/U) was used as a physiological measure of reinnervation and muscle strength
**Blasco et al. **[42]** (2020)**	C57BL/6J mice	Male	1, 14, 24–30 months	N/A	Tibialis anterior (TA) Extensor digitorum longus (EDL) Soleus (Sol) Gracilis (Gra)	• Motor behavioral tests: open-field test • Rotarod and grip strength tests	Wet weight measurement	• Immunohistochemistry for NMJ: area, denervation—assessed by lack of apposition, polyinnervation, fragmentation, terminal sprouting, degree of maturity	Needle electrophysiology: • Nerve conduction tests: Compound muscle action potential (CMAP, M wave) and H-reflex recorded from TA and plantar interossei (PL) muscles with microneedle electrodes • Sensory nerve conduction: Recording electrodes near digital nerves of the fourth toe; stimulation of the sciatic nerve • Parameters: Amplitude (baseline to peak), latency (stimulus to negative peak) • Motor unit number estimation (MUNE) Quantitative electromyography
**Rosenheimer et al. **[57]** (1985)**	F344 rats	Male	10, 28 months	N/A	Extensor digitorum longus (EDL) Diaphragm Soleus	N/A	N/A	N/A	Intracellular recordings of miniature end-plate potentials (MEPPs)
**Padilla et al. **[45]** (2024)**	C57BL/6 mice	Male and female	21–25 months	Ketogenic diet (KD) group Regular chow group	Gastrocnemius Soleus	• Grip strength test • Rotarod performance • Plantarflexion muscle contractility: In vivo contractility system: peak twitch torque and tetanic torque	• Wet weight • Body weight	N/A	Needle electrophysiological assessments: peak-to-peak amplitude of compound muscle action potential (CMAP), single motor unit potential (SMUP) amplitude, repetitive nerve stimulation (RNS), motor unit number estimation (MUNE)
**Ueta et al. **[48]** (2020)**	C57BL/6N mice	Male	24, 24.5, 26.5, and 28 months	Systemic (intravenous) administration of an adeno-associated virus serotype 9 vector encoding human DOK7 (AAV-D7) or empty vector control	Tibialis anterior (TA) Soleus muscle Gastrocnemius Plantarflexor muscles	• Rotarod test: latency to fall • In vivo muscle strength by stimulating plantarflexor: maximal plantarflexion isometric torque	N/A	• Immunohistochemistry of NMJ: areas of presynaptic motor nerve terminals, areas of postsynaptic AChR clusters, cover ratio (nerve terminal area to AChR cluster area), fragmentation of AChR clusters (fragmented if ≥ 5 segments), percentage of denervated NMJs (no presynaptic marker on AChR cluster)	Needle electrophysiology: compound muscle action potentials (CMAPs)
**Alshuaib et al. **[55]** (1991)**	C57BL/6J mice	Male	10, 24 months old	N/A	Soleus	N/A	N/A	N/A	• Intracellular recordings of miniature endplate potentials (MEPPs) • Resting membrane potential
**Smith et al. **[56]** (1988)**	F344 rats	Male	10, 25–27 months	N/A	Diaphragm	N/A	Wet weight/protein ratios	N/A	• Electrophysiology: • Voltage-clamp recordings of spontaneous miniature end-plate currents (m.e.p.c.s) and ACh-induced membrane current fluctuations via microelectrodes: m.e.p.c. amplitudes, time to peak, and decay time constants • AChE activity: radiometric method for AChE forms
**Yamaguchi et al** [35]** (2025)**	C57BL/6J mice	Male	3, 5, 22, 24 months	Aged control (AC): remained sedentary Aged trained (AT) Young mice remained sedentary	Gastrocnemius Plantaris Soleus	• Voluntary grip strength: • Ankle plantar flexion torque: • Muscle stimulation: Percutaneously delivered to the right lower hindlimb using surface electrodes • Nerve stimulation: Delivered to the distal sciatic nerve using needle electrodes • Peak torque and torque after the 100th pulse were analyzed. The ratio of nerve-evoked to muscle-evoked torque was used as a measure of neuromuscular transmission	Wet weight	• Immunohistochemistry of NMJ: • Neurofilament 200 (axons), synaptophysin 1 (nerve terminals), and α-bungarotoxin (α-BTX) (AChRs): • Nerve axon width • Nerve terminal area • AChR area • Number of AChR fragments • Pre- to post-synaptic overlap (ratio of AChR area beneath nerve terminal to total AChR area; overlap < 5% defined as denervated)	Needle electrophysiological assessments: peak-to-peak amplitude of compound muscle action potential (CMAP), single motor unit potential (SMUP) amplitude, repetitive nerve stimulation (RNS), motor unit number estimation (MUNE)
**Alshuaib et al. **[54]** (1990)**	C57BL/6J mice	Male	10, 24 months	N/A	Soleus	N/A	Body weight	N/A	Electrophysiological recording: miniature end plate potentials (mEPPs): Frequency and amplitude and quantal content
**Wilson et al** [53]** (1984)**	Sprague Dawley rats	Male and female	Female rats: Days 28, 42, 56, 84, 168, 364 Male rats: Day 28, 42, 56, and 84	N/A	Sternomastoid	N/A	N/A	Neuromuscular junction and nerve terminal size	Electrophysiological recording: miniature end plate potentials (mEPPs): Frequency and amplitude and quantal content

**Table 3:** Muscle and NMJ morphology and function in preclinical studies

Study	Muscle-related results	Nerve terminal morphology results	Electrophysiology results	Conclusion
**Fahim et al. **[49]**(1997)**	*Young control vs young exercised vs old control vs old exercised* *Muscle mass* • No significant difference in body weight and muscle fiber diameter between all groups *Muscle strength* • Significant lower twitch tension in young control than in old control • Significantly higher twitch tension in young exercised than young control mice • Significantly lower twitch tension in old exercised than old control mice	*Effects of age* • Significantly increase in nerve terminals junctional area, perimeter and extension length in junctional area in 28-month-old unexercised mice compared with 10-month-old controls • The NMJ were larger, more complex, and highly branched with advancing age *Effects of exercise* • In young animals, the 3-month training protocol produced a significantly larger nerve terminal area, perimeter and longer extension lengths • In old animals, GM nerve terminals from old exercised mice had significant smaller areas, shorter perimeters, and shorter extension lengths than those in control old mice	*Effects of age* • MEPP amplitude increased 19% with age • MEPP rise times and one-half decay times were the same in both age groups • No significant changes in resting membrane potential (*E*_m_) with either aging or exercise • There was a significant increase in input resistance (*R*_in_) between 10- and 28-month-old • MEPP frequency decreased 32% with sedentary aging • EPP amplitude significantly increased with age • Calculated quantal content in low-Ca^2+^/high-Mg^2+^ Krebs solution was significantly increased in 28-month-old animals compared with10-month-old animals *Effects of exercise* • Exercise increased the MEPP amplitude in 10-mo-old animals, whereas it decreased MEPP amplitude in 28-month-old animals • Exercise did not affect 10-month-old animals but decreased the *R*_in_ in 28-month-old animals • Exercise decreased MEPP frequencies of 10-month-old mice, increased MEPP frequencies of 28-month-old animals • Exercise increased quantal content in 10-month-old mice, decreased quantal content in 28-month-old animals	• Young NMJ undergo a process of hypertrophy in response to exercise • Endurance exercise resulted in significant reductions in the structure and function of old NMJ • The calculated quantal content confirmed the increased transmitter release capacity of old NMJ • Adding endurance exercise to the aging condition prevented the age-related increase of quantal content
**Chung** **et al. **[37]** (2017)**	• Grip strengths for both forelimbs and all limbs, normalized by body weight, were significantly reduced in the old mice	• Postsynaptic motor endplates of old mice were fragmented and disorganized, whereas those of young mice maintained a pretzel-shaped, branched structure in a well-organized fashion • NMJs in the old mice showed significantly lower occupancy of postsynaptic area by presynaptic terminals • Numerous muscle fibers were observed in the old mice exclusively with AChRs scattered diffusely along the entire fiber without forming an NMJ structure with pre-terminal axons (complete denervation) • The total size of the postsynaptic area was larger in the old mice than in the young mice • No statistical differences in the number of axons per root or per cross-section of ventral roots, or the diameter of ventral root axons, G-ratio between the 2 age groups • Axon diameter frequency histogram shows a Gaussian distribution in the young mice, whereas the old mice show a bimodal distribution of axon diameter with outliers in both directions	Increased SFEMG jitter values were observed in older mice	Dying-back axonal degeneration may be partially responsible for the electrophysiological and strength changes observed with aging
**Iyer** **et al. **[46]** (2021)**	*Muscle mass* • AAV-FST injection resulted in significantly increased gastrocnemius, soleus, triceps brachii muscle mass *Muscle strength* • Tetanic torque demonstrated significant reduction with time (loss of contractility with aging) • Tetanic torque was increased with AAV9-FST treatment, but there was no significant interaction between time and treatment • Tetanic torque normalized to gastrocnemius and soleus weight was not significantly different between treatment groups • A significant interaction between time and treatment for increased twitch torque in the AAV9-FST treated mice • Twitch torque normalized to soleus and gastrocnemius weight was significantly less at endpoint in the AAV9-FST injected mice versus controls	• Presynaptic nerve terminal and bungarotoxin (green) to indicate postsynaptic acetylcholine receptors, revealed a significant improvement in NMJ innervation upon follistatin injection • NMJ fragmentation but found no significant difference between the vehicle and FST groups	• Longitudinal CMAP responses demonstrated significant amplitude losses in both AAV9-FST treatment and vehicle groups over time (loss of amplitudes with aging) • CMAP amplitudes were significantly improved with AAV9-FST treatment when compared to vehicle-injected controls, but there was no significant interaction between time and treatment • SMUP amplitude demonstrated significant change with time • SMUP showed no change with treatment or interaction between time and treatment • MUNE demonstrated significant losses of motor unit numbers over time but no significant change with treatment or interaction between treatment and time • There was no significant difference for CMAP amplitude per gram of gastrocnemius and soleus weight when comparing mice injected with FST versus vehicle • AAV9-FST injected mice showed significantly reduced jitter and blocking percentage compared with Vehicle injected mice at end point	Follistatin overexpression-induced muscle hypertrophy not only increased muscle weight and torque production but also countered age-related degeneration at the neuromuscular junction in mice
**Chung et al. **[43]** (2018)**	• Significant reduction in grip strength of both forelimb and all limbs in aged mice • Standing activities were significantly reduced in aged mice, while walking activity was reduced in the old mice group without significance • NOS is significantly higher in the old mice group	N/A	• Jitter of NMJ transmission was significantly increased in older animals, while CMAP shows no difference between young and old age groups *Correlation* • There was a significant, inverse correlation between single-fiber EMG jitter level and the grip strength of both forelimbs and all limbs • There was a significant, inverse correlation between jitter level and standing activity • Jitter levels didn’t correlate with walking or NOS • CMAP did not correlate with any of grip strength measures, standing activity, walking activity or NOS *Multiple regression analyses between jitter and both grip strengths* • Model explained 63% of variance for forelimb strength and 69% of variance for all limb strength • When age was included in the covariate analysis, jitter did not predict grip strength	Increase in SFEMG jitter level, not reduction in CMAP amplitudes, is associated with reduction in motor function with aging, suggesting that failure in neuromuscular junction plays an important role in early stage of sarcopenia
**Chugh et al. **[44]** (2020)**	• Significant decreases were noted in the aged mice at 20, 24, 27, and 29 months for MUNE, muscle contractility, and frailty index *Correlation* • Significant associations between MUNE, frailty, and blocking on SFEMG in 27- and 29-month-old • Wet weight of the triceps surae muscle was tightly associated with MUNE and frailty index in 27- and 29-month-old	N/A	• Mean jitter and blocking were significantly increased at 27 and 29 months of age • Increased stimulation frequency of 20 and 30 Hz does not significantly increase the ability to detect junctions with blocking as compared with 10 Hz stimulation at 21–30 months of age • Increased frequency of stimulation resulted in higher rates of blocking at 21–30 months of age • Increased MEPC amplitude and decreased MEPC frequencies were observed between 24-month-old and 29-month-old • There was no significant difference in EPC amplitude, or the decrement or facilitation of the EPC	Late NMJ transmission defects imply that modulating NMJ at earlier stages of life may not have relevant functional consequences
**Padilla et al. **[34] **(2021)**	*Muscle mass* • Absolute muscle mass was significantly lower in aged males versus young males but not in aged females versus young females • There were significant reductions of muscle mass when normalized to body mass and comparing across all sexes *Muscle strength* • Hindlimb grip testing revealed significant loss of muscle strength (both absolute and normalized to body mass) between young and aged rats • Forelimb grip testing revealed no difference between young and aged rats when comparing absolute values or normalized values • Increased loss of torque production in aged rats during trains of tetanic stimuli • Increased titanic fade in aged rats during 1-s train of stimuli at 90 and 150Hz	N/A	• The RNS data demonstrated significantly greater reduction of CMAP amplitudes during trains of stimuli in aged versus young rats • SFEMG jitter and blocking was significantly higher in aged rats • MUNE was significantly lower in forelimb and hindlimb muscles in aged versus young rats • SMUPs differed significantly between groups in the hindlimb but not the forelimb *Correlations* • SFEMG jitter and blocking significantly correlated with muscle weight but showed only a trend for normalized hindlimb grip strength, showed significant inverse correlations with CMAP amplitude and MUNE but not SMUP • RNS was significantly correlated with both muscle weight and normalized hindlimb grip strength, SMUP and MUNE but not CMAP • Peak plantarflexion torque (125 Hz stimulation, normalized to body mass) did not correlate significantly with SFEMG jitter but was significantly correlated with both SFEMG NMJ blocking and CMAP amplitude decrement on RNS • Peak tetanic torque at 125 Hz normalized to gastrocnemius mass did not show significant correlation with mean jitter, NMJ blocking or CMAP amplitude decrement on RNS • Tetanic fade at 90 Hz was significantly correlated with SFEMG jitter and blocking and CMAP amplitude decrement on RNS	• These findings provide direct evidence for NMJ dysfunction as a potential mechanism of age-related muscle dysfunction pathogenesis and severity • These findings suggest that NMJ transmission modulation may serve as a target for therapeutic development for age-related loss of physical function
**Ham** **et al** [39]** (2020)**	*Muscle mass* • 30-month-old control mice (30mCON) showed 20–22% loss in muscle mass compared to 10-month young controls • 30-month rapamycin-treated aged mice preserved tibialis anterior (TA) and triceps brachii mass but exacerbated gastrocnemius mass loss *Muscle strength* • Peak tetanic force (extensor digitorum longus and soleus) declined in 30-month-old control mice but was preserved in 30-month rapamycin-treated aged mice • Specific force (force/CSA) improved in rapamycin-treated mice, indicating enhanced muscle quality • Fatigue resistance increased in aged extensor digitorum longus but unaffected by rapamycin	Aged mice (30mCON): • Axon thinning: Reduced axon diameter in EDL and GAS NMJs • Sprouting: Increased presynaptic axon sprouting (compensatory reinnervation) • Postsynaptic Fragmentation: Higher number of fragmented AChR clusters • Reduced AChR Density: Trend toward lower postsynaptic AChR density in EDL Rapamycin-treated mice (30mRM): Rescued NMJ Structure: Normalized axon diameter and sprouting • Reduced AChR cluster fragmentation • Restored AChR density TSCmKO Mice (Muscle-Specific mTORC1 Activation): • Mimicked aged NMJ morphology: Axon thinning, sprouting, and fragmented AChR clusters • 4-week rapamycin treatment reversed these defects	• TSCmKO mice: Increased quantal content (vesicles released per nerve impulse) in TA and SOL NMJs • Aged mice: Similar increases in quantal content (not explicitly measured in 30mCON) • Transmission Fatigue: TSCmKO mice: Rapid rundown of endplate currents (EPCs) during repetitive nerve stimulation (4–15 Hz) • Aged mice: Reduced compound muscle action potential (CMAP) amplitude during repeated stimulation • Aged mice (28–30 months): Nerve-stimulated force in SOL was ~ 75% of direct muscle stimulation • TSCmKO mice: Nerve-stimulated force decreased significantly compared with controls, rescued by rapamycin	• mTORC1 hyperactivity in muscle disrupts postsynaptic AChR stability and presynaptic axon structure, leading to transmission defects • mTORC1 overactivity in muscle fibers drives sarcopenia by destabilizing NMJs, promoting denervation, and inducing inflammatory/ECM remodeling pathways • Rapamycin preserves NMJ integrity, muscle mass, and function
**Pannerec et al. **[36]** (2016)**	*Muscle mass* • Muscle mass (e.g., tibialis anterior (TA)) progressively declined with age, reaching ~ 50% atrophy by 24 months • Forelimb muscles (e.g., biceps brachii) maintained stable muscle mass and fiber cross-sectional area even in old age, and did not show atrophy nor a shift in fiber types • Fast type IIB fibers are most susceptible to age-induced atrophy, seen only in the TA and not in biceps brachii *Muscle strength* • Decrease in gait speed and stride length • Stand time increased for both limbs, but swing time was reduced specifically for hindlimbs	NMJ Fragmentation • Significantly increased with age in hindlimb muscles (e.g., EDL), rising from ~ 20% in adults to ~ 70% in sarcopenic rats • In forelimb muscles (biceps brachii), the proportion of fragmented NMJs remained stable across ages, despite a higher baseline fragmentation due to inherent architectural differences Motoneuron Connectivity • The number of spinal cord motoneurons innervating the gastrocnemius (hindlimb) declined by more than 50% in sarcopenic rats versus adults • Motoneuron numbers for the triceps brachii (forelimb) remained unchanged with age Nerve Molecular Changes • Gene ontology enrichment pointed specifically to cholesterol biosynthesis pathway dysregulation in the sciatic nerve with age • Genes related to neuromuscular function were specifically deregulated in sarcopenia-prone (hindlimb) muscles, not in forelimb muscles • All rate-limiting enzymes of cholesterol biosynthesis were downregulated with age, more so in sciatic than radial nerve. Cholesterol is critical for myelin synthesis; its deficit may underlie neuromuscular deficits • Neurotrophic factors (e.g., Bdnf, Fgf5, p75NTR) were upregulated in aged sciatic nerve, likely as compensatory responses	• Compound Muscle Action Potential (CMAP): amplitude progressively declined with age in hindlimb muscles (gastrocnemius, TA), but was fully preserved in forelimb muscles (biceps brachii, triceps brachii) • This decline in CMAP amplitude correlated tightly with muscle atrophy and functional deficits in the hindlimb, but not forelimb	• Only muscles with impaired neuromuscular transmission, NMJ fragmentation, and motoneuron loss undergo sarcopenia, while those with preserved neuromuscular integrity are spared • Cholesterol biosynthesis dysregulation in the sciatic nerve is identified as an early molecular event upstream of neuromuscular dysfunction and sarcopenia • Local rather than systemic cues determine susceptibility to sarcopenia, as indicated by the regional differences within the same animal or human • Neuromuscular dysfunction is likely a primary driver of sarcopenia, with impaired nerve-muscle interactions setting off a cascade leading to muscle loss
**Banerjee et al. **[60]** (2024)**	No direct age-dependent morphological changes in the muscle fibers	• Synaptic boutons at the neuromuscular junctions (NMJs) shrink and fragment, with the proportion of boutons containing only a single active zone increasing over time • The level of Trio at synaptic terminals declines with age • Knockdown of Trio in adult neurons accelerates synaptic fragmentation and shrinkage • Overexpression of either Drosophila or human Trio preserves synaptic bouton size and prevents fragmentation, even in very old flies. Inducing Trio expression during midlife can halt ongoing synaptic degeneration • The GEF1 (Rac-activating) domain of Trio is essential for this preservation; mutation of GEF1 abolishes protective effects, while GEF2 (Rho-activating) mutation does not • Inhibition of Rac activity increases fragmentation, while activation of Rac can increase bouton size, supporting the role of Trio-Rac signaling in structural maintenance	• Synaptic Rac activity declines by 80% in aged animals, linking reduced Rac signaling to aging • Electrophysiological recordings show that loss of Trio does not affect basic neurotransmission (EJC amplitude, mEJC amplitude/frequency) in young adults, indicating that its primary effect is structural rather than directly on neurotransmitter release • With age, NMJs show decreased ability to sustain high-frequency neurotransmitter release (100-Hz stimulation), with a 56.5% reduction in event amplitude in older animals compared to young • Inhibition of Trio exacerbates this deficit, while overexpression of Trio restores high-frequency neurotransmitter release in middle-aged flies (59.3% increase over controls) • The readily releasable pool (RRP) of vesicles is unchanged by Trio overexpression, suggesting the preservation of synaptic function is due to structural integrity rather than changes in vesicle pool size • Enhanced Trio expression ameliorates synaptic fragmentation and functional deficits in mutants with reduced miniature neurotransmission, again without affecting miniature event frequency or amplitude	• Aging leads to fragmentation and shrinkage of NMJ synaptic boutons, accompanied by diminished motor ability and reduced Rac activity • Trio protein levels at synapses decline with age; this decline is post-translational and not due to reduced mRNA • Knockdown of Trio in adults accelerates synaptic degeneration, while overexpression of Drosophila or human Trio prevents it • Trio’s preservation of synaptic structure depends on its Rac GEF1 activity • Enhanced Trio expression in aging or in models of reduced miniature neurotransmission preserves synaptic structure and function • Trio overexpression delays the age-dependent decline in motor ability, especially in midlife, and maintains the ability of nerve terminals to support high-frequency neurotransmitter release
**Azpurua et al. **[61]** (2018)**	No direct evidence for changes in muscle structure or intrinsic muscle excitability	• No change was observed in the number of synaptic boutons per muscle fiber or in total synaptic volume after dMMP1 overexpression. There was also no evidence of synaptic retraction or die-back	• Acute induction of dMMP1 expression in motor neurons led to significant, reversible reductions in excitatory postsynaptic potentials (EPSPs), indicating reduced neurotransmitter release (quantal content, QC) at the NMJ • The amplitude of miniature EPSPs (mEPSPs), which reflects postsynaptic muscle responsiveness to single vesicles, did not change, suggesting that the effect was presynaptic (i.e., at the nerve terminal) and not due to altered muscle excitability • Neurotransmission and motor function recovered when dMMP1 expression was stopped, indicating that the effect was not due to cell death but to a reversible synaptic dysfunction	• The study concludes that age-dependent upregulation of dMMP1 in Drosophila motor neurons impairs motor function by reversibly disrupting neurotransmitter release at the neuromuscular junction, without causing morphological changes to the nerve terminal or affecting muscle excitability • Inhibition of dMMP1 activity by overexpressing tissue inhibitor of metalloproteinases (dTIMP) delayed age-related motor dysfunction, supporting the idea that dMMP1 acts as an antagonistically pleiotropic gene—beneficial during development but deleterious when re-expressed in aging neurons
**Sugita et al. **[58]** (2021)**	*Muscle mass* • At 23 months, the extensor digitorum longus muscle weight was significantly lower in all aged groups (CONT, MFGM, EX-CONT, EX-MFGM) compared to the young group (YOUNG) • There were no significant differences in muscle weights among the aged intervention groups *Motor Function (Rotarod Test)* • Exercise alone did not significantly improve rotarod performance compared to control diet • Combination of MFGM and exercise (EX-MFGM) significantly increased the time spent on the rotarod compared to the control group	• In young mice, NMJs exhibited a typical pretzel-like morphology with acetylcholine receptors (AChRs) closely apposed to axons • Aged control mice showed fragmented AChRs and partially/fully denervated NMJs • Exercise alone did not significantly reduce the frequency of AChR fragmentation or denervated NMJs, though there was a trend toward fewer fragmented AChRs • The combination of MFGM and exercise (EX-MFGM) significantly decreased both the percentage of fragmented AChRs and denervated NMJs compared to control diet • When intervention began at 18 months (old age), exercise alone only reduced AChR fragmentation but not denervation, whereas MFGM plus exercise significantly reduced both fragmented AChRs and denervated NMJs • MFGM plus exercise increased the expression of Dok-7 Peripheral Nerve Structure • Aging led to a reduction in the number of nerve axons, increased abnormal myelin morphologies (inclusions, outfoldings, tomacular structures), and a higher g-ratio (thinner myelin) • Exercise alone partially attenuated axon loss and improved the g-ratio but did not impact myelin abnormalities • The combination of MFGM and exercise almost completely suppressed age-related loss of nerve axons, myelin abnormalities, and g-ratio increases, indicating robust preservation of nerve structure	Motor nerve conduction velocity (MNCV) • Aging reduced motor nerve conduction velocity • Exercise alone did not improve this decline • Only the combination of MFGM and exercise prevented the age-related decrease in nerve conduction velocity, indicating functional protection of peripheral nerves	• The combination of MFGM intake and exercise does not prevent age-related muscle atrophy but does improve motor coordination and function • Attenuates age-related morphological changes in NMJs, particularly denervation, more effectively than exercise alone • Preserves structure and function of peripheral nerves, reducing age-related axon loss, myelin abnormalities, and slowing of nerve conduction • Its protective effects are seen not only when initiated in midlife but also when started at older ages after NMJ changes have already occurred • These findings suggest MFGM plus exercise as a promising approach to prevent or ameliorate age-related neuromuscular degeneration and functional decline in the elderly
**Kelly** **et al. **[51] **(1986)**	N/A	N/A	• In both young and old soleus, epp amplitude during tetani was maintained at about 45% of baseline, with rapid post-tetanic recovery; in edl, epp amplitude fell more sharply, particularly in old mice, but absolute amplitudes were higher in old muscles • In edl muscles, epp amplitude during a 20-Hz train of 1200 impulses declined steadily to about 20% of control in young mice and even more in old mice; recovery was slower in old mice, but the absolute amplitude was greater in old than young muscles • During longer trains (6000 impulses at 10 Hz) in soleus, epp amplitude decayed to 40–50% in young and 30–40% in old muscle, but recovery was similar, and absolute epp amplitudes were higher in old than young muscle • Use of the false transmitter precursor homocholine (HoCh) showed that in its presence, there was a substantial rundown of epp amplitude during prolonged tetani (10 Hz/6000 pulses), with incomplete recovery, more pronounced in old muscles (down to 13% of control in old, 20% in young) • The rate of replacement of acetylcholine (ACh) by acetyl-HoCh (AHoCh) in released quanta was greater in old than young muscle, with the decay rate constant in old soleus more than twice that in young • The decrease in epp amplitude during tetani in the presence of HoCh was due to decreased quantum size, not fewer quanta released per impulse. Mepp amplitude reductions mirrored those of epp amplitudes	• Despite some greater percentage rundown of epp amplitude during tetani in old animals, the safety factor of neuromuscular transmission was not more compromised in old animals compared to young. Absolute epp amplitudes were greater in old animals, indicating no functional deficit 1 • Old neuromuscular junctions maintained transmitter release during sustained activity by accelerating transmitter turnover and increasing the number of quanta released per impulse. The increased turnover was partly due to a smaller transmitter pool in old nerve terminals • The mechanism of increased transmitter release in aging was not explained by larger or more branched terminals, but rather by changes intrinsic to the nerve terminal, such as increased active zone density or altered calcium handling
**Kelly** **et al. **[52] **(1986)**	N/A	Nerve terminal area and length: • In soleus muscle, the increase in transmitter release (m) with age was not accompanied by an increase in nerve terminal length or area • In extensor digitorum longus, an increase in terminal size was observed, but it was small compared to the increase in release	• Age-related changes in quantal content: In soleus muscles of old mice, the quantal content (m) of evoked transmitter release was nearly doubled compared to young mice • No significant age-related change in quantal content was observed in the diaphragm muscle • Distribution of binomial fitting: The percentage of muscle fibers where the end-plate potential (e.p.p.) amplitudes conformed to binomial statistics varied with muscle type and age: o Soleus: 44% (young), 36% (old) o Diaphragm: 62% (young), 17% (old) • The direct quantal content (m) and the reciprocal of the square of the coefficient of variation of e.p.p. amplitudes were proportional across all fibers, regardless of binomial fit • Lowering stimulus frequency (from 10 to 0.5 Hz) and shortening train length (from 250 to 100 pulses) in young soleus increased binomial fits, but this did not occur in old soleus or diaphragm	• These results highlight the importance of verifying binomial statistical conformity before interpreting parameters like n and p, as non-stationarity and interaction between releases can confound analysis
**Zhang et al. **[40]** (2024)**	• Muscle-Specific Sirt6 Knockout (HSA-Sirt6 cKO) Mice: • Ablation of Sirt6 in skeletal muscle does not affect normal appearance or body weight but results in significant reductions in grip strength and rotarod performance, indicating impaired motor performance	• NMJ structure: In adult mice, NMJs exhibit a continuous pretzel-like structure, but in aged and Sirt6-deficient muscles, NMJs become fragmented and degenerated • Immunofluorescence staining reveals that Sirt6 is enriched in the synaptic region of NMJs. In Sirt6 cKO mice, NMJs begin to fragment at later stages, while innervating motor neuron terminals are preserved but somewhat distorted • Increased expression of AChRγ (a marker of NMJ degeneration) and a higher percentage of centrally nucleated myofibers (indicative of muscle injury and regeneration) are observed in Sirt6-deficient muscles • Localization of dystrophin and YY1: Dystrophin is enriched in the synaptic region, while the repressor YY1 is lower in synaptic compared to non-synaptic regions, supporting the spatial regulation of Dystrophin by Sirt6/YY1 • Sirt6 ablation leads to a notable decrease in Dystrophin mRNA and protein levels in skeletal muscle, while other DGC components remain unchanged. Dystrophin levels are also reduced in aged mice	• Compound muscle action potentials (CMAPs): In control mice, CMAP amplitude remains stable upon repeated nerve stimulation. In HSA-Sirt6 cKO mice, CMAPs display a frequency-dependent reduction in amplitude, indicating impaired neuromuscular transmission • Miniature end plate potentials (mEPPs): Electrophysiological recordings reveal reduced mEPP amplitudes (indicative of impaired postsynaptic assembly) in Sirt6 cKO mice, while mEPP frequency remains unchanged. These defects are not seen in young (1.5-month-old) mice • Effect of NMN Supplementation: • NMN treatment in aged mice increases mono-ADP-ribosylation and ubiquitination of YY1, enhances Dystrophin protein levels, reduces NMJ fragmentation, and improves CMAP amplitudes as well as motor behavior (grip strength and rotarod performance). These effects are absent in Sirt6-deficient mice, indicating the necessity of Sirt6 for NMN’s beneficial action	• Sirt6 is required for NMJ maintenance and motor function in aging muscle, primarily by regulating Dystrophin expression. Mechanistically, Sirt6 mono-ADP-ribosylates the Dystrophin transcriptional repressor YY1, which releases YY1 from the Dystrophin promoter and promotes its degradation via the SMURF2 E3 ligase pathway • Supplementation with NMN, a precursor of NAD +, enhances Sirt6-mediated mono-ADP-ribosylation of YY1, delays NMJ degeneration, and improves motor function in aged mice. Targeting the Sirt6/YY1/Dystrophin axis is proposed as a potential therapeutic strategy to combat age-related decline in NMJ structure and motor function
**Zhao et al** [41]** (2018)**	• Muscle atrophy and regeneration: In aged mice, muscle fibers were smaller and had a higher number of centrally localized nuclei, which is a sign of muscle regeneration due to degeneration. These age-associated changes were diminished by muscle-specific LRP4 expression in transgenic mice, as well as by AAV9-mediated overexpression of SGα (sarcoglycan alpha). Both interventions led to increased muscle fiber cross-sectional area and reduced central nuclei, indicating improved muscle health and reduced degeneration/regeneration cycles • Muscle Force: Aged mice exhibited reduced twitch and tetanic muscle force. Flag-Lrp4 transgenic mice and aged mice treated with AAV9-SGα-GFP showed increased twitch and tetanic forces, indicating improved muscle function and strength	• NMJ fragmentation and denervation: In aged mice (24 months), there was a significant increase in fragmented acetylcholine receptor (AChR) clusters and denervation at the neuromuscular junction (NMJ) compared to young mice (3 months). Only about 50% of endplates were fully innervated in aged mice, with the rest being partially or fully denervated and highly fragmented • Rescue by LRP4 and SGα Expression: Transgenic expression of LRP4 or AAV9-mediated overexpression of SGα in aged mice increased the percentage of fully innervated endplates (from ~ 51 to ~ 72% with LRP4, and from ~ 49 to ~ 70% with SGα), reduced the number and percentage of fragmented NMJs, and increased AChR cluster intensity (by 48% with LRP4 and ~ 53% with SGα)	• Compound muscle action potentials (CMAPs): Aged mice showed a frequency-dependent reduction in CMAP amplitudes, especially at high stimulation frequencies (20 and 40 Hz), indicating compromised neuromuscular transmission. This reduction in CMAPs was diminished in both Flag-Lrp4 transgenic mice and AAV9-SGα-treated mice • Miniature endplate potentials (mEPPs): mEPP amplitudes were reduced in aged mice, consistent with impaired synaptic function. This reduction was ameliorated by LRP4 or SGα overexpression, while mEPP frequency remained comparable across groups	• Age-related NMJ decline is associated with reduced LRP4 protein levels (but not mRNA), likely due to increased proteasome-mediated degradation and ubiquitination of LRP4. Sarcoglycan alpha (SGα) interacts with LRP4 and stabilizes it, preventing degradation • Increasing LRP4 or SGα expression in muscle can alleviate NMJ fragmentation, denervation, impaired neuromuscular transmission, and muscle atrophy in aged mice. The results suggest that stabilizing LRP4 via SGα or enhancing agrin-LRP4-MuSK signaling could be a promising therapeutic strategy for age-related NMJ decline and sarcopenia
**Kelly** **et al. **[50] **(1983)**	• Age-related changes: E.d.l. and soleus muscles showed significant age-related changes in synaptic transmission, while the diaphragm was relatively unaffected • No significant atrophy was observed in muscles despite functional changes, suggesting the changes were not secondary to muscle disuse or degeneration	• Vesicle changes: Old mouse e.d.l. and soleus muscles showed a depletion of synaptic vesicles and an increase in coated vesicles. These changes are also observed after prolonged tetanic stimulation in younger animals • Profound loss of synaptic vesicles has been noted in neuromuscular junctions of old mice, which could influence transmitter release • Compensatory changes: The increase in quantal content (i.e., more neurotransmitter released per impulse) with age is interpreted as a compensatory, rather than degenerative, response to maintain synaptic efficacy in the face of morphological changes	• End-plate potentials (e.p.p.s): E.p.p. amplitude increased with age in e.d.l. and soleus but not in the diaphragm • The increase in e.p.p. amplitude in e.d.l. occurred between 20 and 28 months, while in soleus it occurred between 28 and 31 months • Twitch response and Mg block: Resistance to Mg block (i.e., less depression of muscle twitch in low Ca/high Mg solution) increased with age, appearing between 15 and 19 months in both e.d.l. and soleus • In CFW mice, e.d.l. muscles from 25-month-old animals were more resistant to Mg block than those from 7- to 8-month-old animals • Quantal content and m.e.p.p.: The increased e.p.p. amplitude in old soleus were due to increased quantum content, not changes in miniature end-plate potential (m.e.p.p.) amplitude • In old soleus, m.e.p.p. frequency was not reduced by low Ca/high Mg, unlike in young soleus or diaphragm, indicating decreased sensitivity of transmitter release to external Ca/Mg in aged muscles • Timing of changes: Most changes occurred between 15 and 26 months, with e.d.l. changes preceding those in soleus. Maximum age-related changes occurred at 26 months, with a decline by 32 months	• Age-related changes in synaptic transmission begin in midlife and manifest more rapidly in e.d.l. than in soleus, possibly due to differences in muscle type or usage patterns • These changes occur before significant mortality or organ pathology, suggesting they are an early and intrinsic sign of neuromuscular aging, not secondary to general health decline • The decreased sensitivity of transmitter release to low Ca/high Mg is an early marker of altered synaptic transmission in aging muscle • The increase in quantal content with age is likely compensatory, possibly in response to subclinical changes in muscle activity, contractile strength, or nerve terminal health • The temporal progression of these changes is relatively abrupt and confined to a limited age window, which may aid in identifying the underlying mechanisms
**Hu et al. **[59]** (2023)**	Muscle integrity and aging: • VPS-34 is expressed in multiple *C. elegans* tissues, including muscles, neurons, and intestine, with highest expression in neurons. Loss of muscle integrity during aging is characterized by increased mitochondrial fragmentation • In vps-34(qx546) mutants, aged worms show significantly reduced mitochondrial fragmentation in body wall muscles compared to wild type, indicating improved muscle integrity • Overexpression of vps-34 in muscles (but not neurons) of qx546 worms restores the fragmentation to wild type levels, suggesting that VPS-34 regulates muscle integrity cell—autonomously in muscle cells • Partial inhibition of VPS-34 via genetics or the inhibitor SAR405 also rescues age-related lysosome dysfunction in the epidermis and increases relative intestinal width in aged worms, though it does not affect the age-related decline in pharyngeal pumping. In aged mice, SAR405 treatment increases the cross-sectional area of muscle fibers, ameliorates muscle atrophy, and improves mitochondrial function in muscle (as shown by increased succinate dehydrogenase staining)	Synapse Number: Partial inhibition of VPS-34 does not affect the number of synapses in aged neurons. Quantification using GFP-tagged synaptic vesicle proteins (RAB-3 and SNB-1) in vps-34 RNAi and vps-34(qx546) animals shows no significant changes in synapse number versus wild type at both young and aged stages	• Synaptic Transmission: The frequency of endogenous miniature postsynaptic currents (mPSC) declines by 70% with age. In vps-34(qx546) mutants, this decline is prevented—aged mutants have mPSC frequencies similar to young wild-type animals. Overexpression of vps-34 in motor neurons of qx546 mutants reverses this improvement • Pharmacological Inhibition: SAR405 treatment of aged wild-type worms significantly enhances mPSC frequency and amplitude, similar to the genetic results; SAR405 has no effect in vps-34(qx546) mutants, confirming its specificity • Downstream Mechanisms: The improvement in synaptic transmission due to vps-34 inhibition requires key exocytosis regulators (unc-13, unc-18, snt-1)	• Partial inhibition of VPS-34—either genetically or pharmacologically—simultaneously improves muscle integrity and enhances synaptic transmission during aging, without deleterious effects on development or lifespan • VPS-34 is an evolutionarily conserved, actionable target to delay motor aging and prolong health span in both *C. elegans* and mice. Pharmacological inhibition by SAR405 offers a promising strategy for improving motor function and muscle health during aging
**Zitman et al. **[86]** (2011)**	• Muscle Strength: Grip strength testing showed that muscle power, when normalized for body weight, was similar among wild-type (WT), GM2s-KO, and GD3s-KO mice • Absolute force was higher in GD3s-KO mice than WT, paralleling higher body weight • There was no evidence of muscle weakness in aged GM2s-KO or GD3s-KO mice despite observed motor deficits in GM2s-KO mice	N/A	• Miniature Endplate Potentials (MEPPs): MEPP frequency was significantly higher in GD3s-KO NMJs compared to WT • MEPP amplitude was larger in GD3s-KO NMJs than in WT or GM2s-KO • GD3s-KO mice showed shorter MEPP rise time and smaller halfwidth compared to WT and GM2s-KO • Evoked Endplate Potentials (EPPs): EPP amplitudes at 0.3 Hz stimulation were similar across all genotypes (22–24 mV) • Quantal content was similar in all groups • EPPs in GD3s-KO mice had shorter rise times and halfwidths • High-frequency (40 Hz) stimulation caused slightly more pronounced rundown in GM2s-KO NMJs compared to WT • No significant differences in rundown at 3-Hz stimulation • Hypertonic Shock and K⁺-Induced Release: No differences among genotypes in ACh release induced by hypertonic medium or increased extracellular K⁺ • Temperature and Ca^2^⁺ dependency: EPP amplitude and frequency differences were most notable at 25°C • No major differences in EPP amplitude or quantal content across temperatures (17–35°C) • GD3s-KO NMJs showed less steep Ca^2^⁺-dependency for ACh release and higher release at low Ca^2^⁺ (0.2 mM) • EPPs and MEPPs in GD3s-KO NMJs remained faster than WT or GM2s-KO at all Ca^2^⁺ concentrations	• Aged GM2s-KO and GD3s-KO mice show only subtle changes in neuromuscular synaptic transmission compared to WT, despite progressive motor deficits in GM2s-KO • Transmission at NMJs remains successful in aged ganglioside-deficient mice, and muscle weakness is not observed • The mutual redundancy of gangliosides in supporting presynaptic function appears adequate even with aging • Slight changes in synaptic kinetics in GD3s-KO mice may suggest a role for O- and/or a-series gangliosides in postsynaptic acetylcholine receptor function, but these changes do not threaten neuromuscular transmission • It is highly unlikely that NMJ transmission failure contributes to the motor defects in aged GM2s-KO mice; deficits are more likely due to peripheral sensory, proximal motor nerve, or CNS dysfunction
**Miyamaru n et al** [47]** (2012)**	• In aged rats where the recurrent laryngeal nerve (RLN) was simply transected (DNV group), the area of the entire thyroarytenoid (TA) muscle and the individual muscle fibers on the treated side were smaller than those on the untreated side, indicating muscle atrophy due to denervation • In aged rats that received nerve-muscle pedicle (NMP) implantation after RLN transection (NMP group), the area of the entire TA muscle and the individual muscle fibers on the treated side were similar to those on the untreated side, indicating preservation or recovery of muscle mass • The treated/untreated (T/U) ratios for both the entire TA muscle area and individual muscle fiber area were significantly higher in the aged NMP groups than in the aged DNV groups • The T/U ratio of the entire TA muscle area in the aged NMP10 group was lower than in the young NMP10 group, but by 20 weeks (aged NMP20) this difference was not significant. The T/U ratios for individual muscle fiber area were similar between aged and young NMP groups	• In aged DNV models, the number of synaptophysin-positive nerve terminals (NTs) was lower on the treated side compared to the untreated side, while the number of acetylcholine receptors (AChRs) was not reduced • In aged NMP groups, both NTs and AChRs were present in nearly equal numbers on treated and untreated sides • The T/U ratio of NTs was significantly higher in aged NMP groups compared to aged DNV groups; similarly, the NT/AChR ratio was also significantly higher in aged NMP groups, suggesting better re-establishment of neuromuscular junctions with NMP implantation • No significant differences were found between aged and young NMP groups for T/U ratios of NTs, AChRs, or NT/AChR ratio	• Upon stimulation of the transferred ansa cervicalis nerve, evoked muscle action potentials (MAPs) were observed in all NMP animals (aged and young). No MAPs were seen after cutting the transferred nerve, confirming the specificity of reinnervation • Stimulation of the proximal end of the transected RLN did not elicit muscle activity, indicating no detectable reinnervation via the original RLN • The T/U ratios of MAPs in aged NMP groups were not significantly different from those in the young NMP group	• NMP implantation is effective in recovering the atrophic changes of the denervated TA muscle in aged rats • The method restores both muscle mass and neuromuscular junctions and results in functional reinnervation as evidenced by electrophysiological testing • The effectiveness of the NMP method in aged rats is almost the same as that in young animals • However, the effectiveness may decrease with longer periods of denervation, as shown by a decline in AChRs with prolonged denervation
**Blasco et al. **[42]** (2020)**	• Muscle mass: No significant difference in wet muscle weights between adult and old mice, though some reduction was noted in the soleus (Sol) and extensor digitorum longus (EDL) muscles • No significant differences in average myofiber size across muscles, but all old muscles showed increased connective tissue, especially EDL • Increased presence of lipofuscin (age pigment) aggregates in myofibers of old muscles • Satellite Cells (SCs): Significant reduction in Pax7-positive SCs (muscle-resident stem cells) in old Sol and EDL muscles • Fiber type composition: Old distal muscles (TA, EDL, Sol) had fewer type 2B fibers and more type 2 A fibers; Gra showed a decrease in type 2 A fibers and an increase in type 2B fibers • Muscle regeneration: Increased numbers of fibers with central nuclei, suggesting ongoing regeneration to compensate for persistent degeneration	• Structural changes: Old muscles exhibited NMJs with partial or complete denervation, polyinnervation, increased terminal branching, sprouting, larger and fragmented endplates • Extrasynaptic acetylcholine receptor (AChR) clusters (indicative of reactive synaptogenesis) were seen along myofibers in old muscles • Molecular Markers: Increased expression of calcitonin gene-related peptide (CGRP), growth associated protein 43, agrin, fibroblast growth factor binding protein 1 (FGFBP1) and transforming growth factor-β1 (TGF-β1)	• Nerve conduction and muscle response: Old mice showed reduced nerve conduction velocity (increased latency time) in both motor and sensory nerves • Compound muscle action potential (CMAP) amplitude was reduced in old mice, especially in distal plantar muscles; this was due to a slight decrease in both the number and size of motor units • Sensory compound nerve action potential (CNAP) recorded in the toes did not significantly decrease, suggesting motor decline is partly related to muscle loss and compressive forces on the foot sole • Motor reflex wave latency (spinal stretch reflex) was longer in old mice (PL muscle), but mean amplitude was unchanged. H/M ratio was higher, likely reflecting compensatory mechanisms • Motor unit number estimation (MUNE): Mild reduction in MUNE in old muscles, consistent with the absence of significant motoneuron degeneration	• Ageing in C57BL/6J mice is not accompanied by significant motoneuron (MN) death in the lumbar spinal cord. Instead, there is a marked loss of excitatory cholinergic and glutamatergic inputs to MNs (deafferentation), coupled with prominent microgliosis and astrogliosis around aged MNs • Old muscles show signs of denervation and reinnervation (polyinnervation, terminal sprouting, fragmented endplates), along with increased expression of molecules associated with plasticity and NMJ maintenance • All hindlimb muscles—regardless of fiber type or location—display features of degeneration and regeneration, with variable severity likely influenced more by muscle function and activity than by topography or fiber composition • MN dysfunction and synaptic alterations, rather than MN loss, underlie age-related motor impairment and sarcopenia. This supports the idea that targeting synaptic maintenance and muscle regeneration could be effective strategies to combat sarcopenia
**Rosenheimer et al. **[57]** (1985)**	• Nerve terminals innervating the EDL had the fastest glucose uptake, followed by diaphragm and then soleus. The difference was statistically significant between EDL and soleus • With aging (comparison between 10-month and 28-month-old rats), there was a tendency for slower rates of glucose uptake in all three muscles, though this did not reach statistical significance due to variability • The study suggests that glucose entry is slowest into cells with the greatest oxidative potential (soleus > diaphragm > EDL), possibly reflecting their differing energy demands and metabolic profiles • The findings are consistent with previous reports that muscle fibers and the nerve terminals that innervate them show metabolic differences corresponding to fiber type	N/A	• Glucose uptake was assessed indirectly by recording miniature end-plate potentials (MEPPs) at the neuromuscular junction. Addition of glucose increased MEPP frequency (due to hypertonic conditions), and the rate at which MEPP frequency returned to baseline reflected the rate of glucose uptake • Increasing the osmolarity of the bathing saline with d-glucose resulted in an initial sharp increase in MEPP frequency, followed by a steady decline as osmotic equilibrium was re-established • The time constant of MEPP decay (representing glucose uptake) was fastest in EDL, intermediate in diaphragm, and slowest in soleus • Experiments with 2-deoxy-d-glucose (a non-metabolized glucose analog) produced similar results, indicating that the changes in MEPP rate were due to glucose uptake, not metabolism • l-Glucose (which does not use the carrier-mediated uptake) did not produce the usual decline in MEPP frequency, supporting the specificity of the carrier-mediated process • The technique requires high glucose concentrations to produce measurable changes in MEPP frequency, and it is noted that age-related changes in high-affinity glucose transporters (which cannot be assayed electrophysiologically) may be missed	• Glucose uptake into nerve terminals varies by muscle fiber type, occurring fastest in glycolytic (EDL) and slowest in oxidative (soleus) muscles • There is a trend toward decreased rates of glucose uptake with age in all three muscle types, though this was not statistically significant due to variability • Nerve terminals with higher oxidative enzyme activity (typically those innervating oxidative muscle fibers) take up glucose more slowly, possibly reflecting their greater reliance on oxidative metabolism as opposed to glycolysis • If age-related decreases in glucose uptake are real, they may reflect an increased oxidative character of nerve terminals with aging, similar to changes observed in muscle fibers themselves • The electrophysiological method used is limited by the high glucose concentrations required and may not detect changes in high-affinity uptake systems
**Padilla et al. **[45]** (2024)**	• Body mass: Mice on the ketogenic diet (KD) did not show a statistically significant increase in body mass compared to controls, though there was a moderate effect size (η^2^ = 0.422; *p* = 0.0882) • Muscle strength: Mice had significantly improved hindlimb grip strength (36% increase in KD vs. 3% in controls) and all limb grip strength (6% increase in KD vs. 5% decrease in controls) over 10 weeks (*p* = 0.0001–0.0005 for diet effect) • Motor performance: Mice showed a 78% improvement in rotarod latency to fall (motor stamina/coordination) compared to a 48% increase in controls (*p* = 0.0126; time × diet, *p* = 0.0021) • Muscle contractility: No significant differences were found in muscle contractility (plantarflexion tetanic torque) between KD and control groups (*p* = 0.5248) • Muscle mass: No significant differences in wet weights of gastrocnemius or soleus muscles, nor when normalized to body mass (*p* > 0.1 for all comparisons)	N/A	• Motor unit number estimation (MUNE): KD mice showed a 16% increase in MUNE at 10 weeks, while controls had a 20% reduction. The diet effect was significant (*p* = 0.0465; time × diet, *p* = 0.0064), indicating improved or preserved motor unit connectivity in aged mice fed a KD • Repetitive nerve stimulation (RNS): No significant differences in neuromuscular junction transmission efficacy (as measured by RNS at 50 Hz) between groups (*p* = 0.3562; time × diet, *p* = 0.9871)	• The ketogenic diet improved neuromuscular and motor function in aged mice, as evidenced by increased muscle strength, improved motor performance, and a higher number of functional motor units (MUNE), without affecting muscle contractility, muscle mass, or NMJ transmission efficacy • KD may help improve or maintain motor function and neuromuscular integrity during aging, though the underlying mechanisms—especially at the level of nerve terminal morphology—require further investigation
**Ueta et al. **[48]** (2020)**	• Muscle strength: DOK7 gene therapy (via AAV-D7) significantly increased muscle strength in aged mice, as measured by in vivo twitch force of the hindlimb muscle after electrical stimulation. Specifically, AAV-D7-treated 28-month-old mice showed significantly higher muscle strength compared to control (AAV-ø-treated) mice. Before treatment at 24 months, there was no difference between groups, but after treatment, muscle strength in AAV-D7 mice increased to 132% of pre-dose values, while AAV-ø mice decreased to 83% • Muscle mass (Myofiber cross-sectional area, CSA): Despite the increase in muscle strength, DOK7 gene therapy did not significantly increase the CSA of tibialis anterior (TA) muscle fibers compared to control aged mice. Both AAV-D7 and AAV-ø groups had reduced CSA compared to young (4-month-old) mice, but there was no significant difference between the treated and control aged groups 6–7 • Body weight: No significant difference in body weight was observed between AAV-D7 and AAV-ø groups during the testing period, suggesting the functional improvements were not due to changes in overall body mass	• NMJ size: AAV-D7 treatment significantly increased both the postsynaptic area of acetylcholine receptor (AChR) clusters and the presynaptic area of synapsin-1-positive motor nerve terminals at NMJs in aged mice, indicating NMJ enlargement • NMJ denervation: DOK7 gene therapy reduced the percentage of denervated NMJs in aged mice. Four months after treatment, AAV-D7-treated 28-month-old mice had a much lower percentage of denervated NMJs than AAV-ø controls and even non-treated 24-month-old mice, suggesting enhanced NMJ reinnervation • AChR cluster fragmentation: AAV-D7 treatment did not significantly affect fragmentation of AChR clusters, a structural hallmark of NMJ aging. Fragmentation levels remained similar between treated and control aged mice • Cover ratio: The ratio of presynaptic nerve terminal coverage of AChR clusters was not changed significantly by DOK7 gene therapy • Muscle types: Similar effects (increased NMJ size, reduced denervation, no significant change in fragmentation or cover ratio) were observed in both fast-twitch (TA) and slow-twitch (soleus) muscles	Compound muscle action potentials (CMAPs): Aged mice (28 months) treated with DOK7 gene therapy showed a significant increase in the maximal amplitude of CMAPs compared to AAV-ø controls and even to non-treated 24-month-old mice. This suggests improved neuromuscular transmission and functional connectivity at the NMJ	DOK7 gene therapy in aged mice activates MuSK, enlarges NMJs, suppresses NMJ denervation, increases CMAP amplitudes, and improves both muscle strength and motor function. These benefits occurred without inducing detectable muscle hypertrophy, suggesting improved NMJ innervation and possibly enhanced mechanical properties of muscle fibers. The results establish proof of principle that enhancing NMJ innervation (e.g., via DOK7 gene therapy) could be a promising approach to ameliorate age-related motor dysfunction, although further study is needed to clarify the precise mechanisms and to address potential limitations for clinical translation
**Alshuaib et al. **[55]** (1991)**	N/A	N/A	• MEPP frequency (miniature end-plate potentials): In normal K + (5 mM) with 0.5 mM Ca, MEPP frequency was lower in old mice (1.1 Hz) than in young mice (1.5 Hz) • In high K + (15 mM) with 0.5 mM Ca, MEPP frequency was significantly higher in old mice (4.7 Hz) compared to young mice (2.6 Hz) • In high K + with 0 Ca (EGTA), MEPP frequency was again lower in old mice (0.8 Hz) than in young mice (1.3 Hz), indicating the increased frequency in high Ca is due to Ca influx, not just depolarization • Increasing extracellular Ca in high K + conditions progressively raised MEPP frequency, with old animals showing much higher frequencies than young at each Ca concentration (1.5, 2.5 mM) • MEPP frequency dependence on Ca: Log–log plots showed the slope of the relationship between Ca concentration and MEPP frequency decreased as Ca increased, with similar sensitivity (slope) in both young and old animals (1.9 vs. 2.0 for 0.5–1.5 mM Ca; 0.9 vs. 0.7 for 1.5–2.5 mM Ca) • Oscillations in MEPP frequency: Periodic oscillations in MEPP frequency were observed in both age groups • In 2.5 mM Ca-high K + solution, old animals had higher MEPP frequencies and shorter oscillation periods (old, 1.3 s; young, 2.1 s) • In Ca-EGTA-high K + solution, old animals had lower MEPP frequencies and longer oscillation periods (old, 4.6 s; young, 2.9 s) • For both age groups, higher Ca led to higher MEPP frequency and shorter oscillation periods	• Resting MEPP frequency is lower at old than at young terminals under normal conditions, but when depolarized and exposed to increasing Ca, old terminals show much higher frequencies than young • Voltage-dependent Ca entry (i.e., Ca influx through Ca channels) increases with aging at the soleus nerve terminal, likely due to an increased number or altered properties of Ca channels • The sensitivity of the transmitter release process to Ca is not altered with age, suggesting the increased release is due to greater Ca entry rather than changes in the release machinery itself • Oscillations in MEPP frequency reflect intraterminal Ca dynamics, with aging affecting both the amplitude and period of these oscillations • Altered Ca homeostasis, specifically increased Ca influx, is a key feature of neuromuscular aging and may underlie both functional plasticity and degeneration observed in aging nerve terminals
**Smith et al. **[56]** (1988)**	• Protein content and muscle weight do not differ significantly with age in the diaphragm muscle	Morphological changes observed in aged rodents’ neuromuscular junctions are similar to those seen after long-term AChE inhibition, including: • Increased numbers of motor nerve terminals • Widened synaptic clefts • Abnormal postsynaptic folds • Subsynaptic myopathy	• Miniature end-plate currents (m.e.p.c.s): m.e.p.c.s were recorded under voltage-clamp before and after AChE inhibition. Inhibition (using echothiopate or methanesulfonyl fluoride) caused increased amplitude and prolonged decay time constants in both age groups, but the magnitude of these changes was larger in older animals • Despite the biochemical increase in AChE activity, less ACh was hydrolyzed in older animals, as indicated by calculations of the fraction of ACh hydrolyzed at a common time point (0.5 ms after onset); this fraction was lower in aged animals • The effect of AChE inhibition on end-plate currents was less in rat than in frog, suggesting species differences in synaptic architecture and ACh clearance mechanisms • The increased decay time of m.e.p.c.s after AChE inhibition in aged rats is likely due to an expanded field of postsynaptic ACh receptors, not a change in junctional AChE activity • Single-channel properties: No significant changes in mean channel open time or single-channel conductance were found with age or after AChE inhibition	• Total AChE activity at the rat diaphragm neuromuscular junction increases with age, especially in certain molecular forms and mainly in extrajunctional regions • Despite increased total AChE activity, there is no significant increase (and possibly a decrease) in junctional AChE density with age. The proportion of ACh hydrolyzed at the synapse decreases in aged rats, but this does not alter end-plate current properties under normal conditions, likely due to a compensatory increase in postsynaptic ACh receptor field size • The observed physiological and morphological changes in aged rats’ neuromuscular junctions may thus represent adaptations to increased ACh leakage and altered synaptic function with aging
**Yamaguchi et al** [35]** (2025)**	• Muscle size and grip strength: Before intervention, young mice had significantly lower body weight than both aged control (AC) and aged trained (AT) mice. After 2 months, the body weight of young mice increased, eliminating group differences • Grip strength tended to be higher in young mice compared to AC and AT before intervention. After intervention, grip strength was significantly lower in AC compared to both young and AT mice, indicating that endurance training prevented the age-related decline in muscle strength • Wet weight of the gastrocnemius muscle showed no group difference; plantaris muscle weight was lower in AC vs. young mice; soleus muscle weight was lower in both AC and AT vs. young mice. Total wet weight of all three muscles was lower in AC than in young mice • Myofiber composition and size: No significant group effect on myofiber number in either plantaris or soleus • No significant group effect on myofiber composition (type I, IIa, IIb/IIx) in plantaris • In soleus, AC mice had a higher proportion of type I fibers and a lower proportion of type IIb/IIx than young mice, reflecting an age-related shift toward a slower phenotype. Myofiber size was not significantly different between groups • Effect of endurance training: Endurance training prevented most age-related changes in muscle strength but did not significantly alter myofiber composition compared to AC, although the composition in AT was closer to young mice	• NMJ denervation and morphology: In the plantaris muscle, the proportion of denervated NMJs (overlap < 5%) was significantly higher in AC than in young and AT mice • Among innervated NMJs: No significant group effect on axon width • AC mice had smaller nerve terminal area than young and AT mice • No group effect on AChR area or number of AChR fragments • Degree of synaptic overlap was lower in AC than in young and AT mice • Fiber-type specific denervation (Soleus): Denervation was more frequent in fast-twitch fibers in AC mice than young and AT mice, but not in slow-twitch fibers • In AC mice, nerve axon width and nerve terminal area were smaller in both fast- and slow-twitch fibers, but more so in fast-twitch • Synaptic overlap was lower in AC than in young and AT mice for both fiber types, but greater in slow-twitch than fast-twitch fibers • Effect of endurance training: Endurance training restored nerve terminal area and synaptic overlap, and reduced denervated NMJ proportion in aged mice	• Neuromuscular transmission (torque measurements): No group effect on peak nerve (NT) or muscle (MT) stimulation torque • The ratio of NT to MT (an index of neuromuscular transmission) was lower in AC than in young and AT mice at high-frequency (150, 200 Hz) stimulation, especially in the late phase (after 100 pulses) • The NT/MT ratio at high frequencies correlated with voluntary grip strength • Motor unit number estimation (MUNE): MUNE was lower in AC compared to young and AT, indicating age-related loss of functional motor units, which was partially restored by endurance training • No group difference in compound muscle action potential (CMAP) amplitude, but single motor unit potential (SMUP) amplitude was higher in AC vs. AT	• Age-related changes at the NMJ (decreased synaptic overlap, nerve terminal area, and increased denervation—especially of fast-twitch fibers) lead to impaired neuromuscular transmission and a shift toward slower muscle fiber types, contributing to muscle weakness • Endurance training prevented or ameliorated much of the age-related deterioration in NMJ structure and neuromuscular transmission, preserving muscle strength and partially maintaining myofiber composition • The degree of pre- to post-synaptic overlap at the NMJ is a strong correlate of neuromuscular transmission efficiency and, by extension, voluntary muscle strength
**Alshuaib et al. **[54]** (1990)**	• No significant difference in mean body weights between young and old groups	N/A	• Quantal content: Quantal content (mean number of ACh quanta released per nerve impulse) was significantly higher in old mice (0.58 ± 0.13) than in young mice (0.41 ± 0.09), a 41.5% increase • Post-tetanic potentiation (PTP) and MEPP frequency: In Ca^2^⁺-containing Krebs solution—both young and old terminals showed increased MEPP (miniature endplate potential) frequency during and after tetanus • Old terminals had greater tetanic MEPP frequency and a longer decay time (post-tetanic potentiation (PTP)) than young terminals • Time constants of decay for augmentation (TA) and potentiation (TP) were both significantly longer in old mice (TA, 10.3 ± 1.0 s; TP, 195.3 ± 5.4 s) compared to young (TA, 7.0 ± 0.7 s; TP, 78.8 ± 6.6 s) • In Ca^2^⁺-free/EGTA Krebs solution: No significant difference in PTP properties between young and old terminals, indicating age differences depend on Ca^2^⁺ influx, not on intracellular Ca^2^⁺ buffering/clearance • MEPP frequencies and PTP decays were much lower in EGTA Krebs, and the time constant for potentiation did not differ between ages • Oscillations and consistency: Periodic oscillations in post-tetanic MEPP frequency, presumably reflecting changes in intraterminal free Ca^2^⁺, had a longer period in young (27.9 ± 4.3 s) than old (14.6 ± 2.2 s) terminals • The time course of PTP was reproducible at the same nerve terminal	• Mechanism underlying increased transmitter release with aging is primarily an increase in Ca^2^⁺ influx, possibly due to changes in the number or properties of presynaptic Ca^2^⁺ channels, rather than changes in Ca^2^⁺ clearance or sensitivity of the release machinery
**Wilson et al** [53]** (1984)**	N/A	• Junctional size increase: In the sternomastoid muscle of female rats, neuromuscular junctions increase in size up to about 250 days of age, but most of this increase occurs by 44 days • Relationship to release: The increase in neuromuscular junction size is associated with an increase in quantal release • Sex differences: No significant difference in the properties of the nerve terminal between the sexes	Quantal content of first EPP: 28-day-old female rats—37 ± 3 quanta • 28-day-old male rats—33 ± 3 quanta • At 42 days of age, quantal release values significantly increased in both sexes (females, 59 ± 4; males, 50 ± 4) • No significant increase in quantal release after 42 days, up to 364 days Plateau EPPs (mobilization rate): • In 28-day-old females—23 ± 1 quanta at 50 Hz (mobilization capability, 1150 quanta/s) • In 28-day-old males—22 ± 2 quanta (1100 quanta/s) • At 42 days: significant increase in mobilization rate (females, 41 ± 3 quanta, 2050 quanta/s; males, 34 ± 2 quanta, 1700 quanta/s) • No significant increase in mobilization rate after 42 days Mepps: Age-related increase in MEPP frequency and decrease in amplitude were observed and correlated with neuromuscular junction size and muscle fiber diameter	• The neuromuscular junction in the rat diaphragm matures physiologically by 6 weeks of age (42 days) and remains stable through the first year of life (364 days) • Major changes in quantal content, statistical store, and mobilization rate only occur between 28 and 42 days of age • After 42 days, increases are minor and statistically insignificant, indicating functional maturity • No significant sex differences in nerve terminal properties despite body size differences • The findings are reconciled with morphological studies showing junctional size increases primarily before 44 days, with only modest increases thereafter • No further significant changes in neuromuscular transmission would occur until advanced age is reached

### Clinical studies

A variety of electromyographic (EMG) techniques were employed across studies to comprehensively assess neuromuscular activation, motor unit properties, and NMJ transmission. Surface EMG was most commonly used to measure global muscle activation patterns, including maximal voluntary contraction (MVC) amplitude, integrated EMG, the rate of EMG rise, and burst characteristics during dynamic and isometric tasks [5–16]. Multi-channel surface EMG enabled the identification and tracking of individual motor unit firing rates during ramp contractions [17]. Intramuscular EMG (iEMG) provided detailed analysis of MUP morphology, firing rates, area, duration, and complexity, as well as near-fiber measures such as jiggle and jitter, which are sensitive indicators of NMJ transmission stability and motor unit remodeling [10, 18–23]. SFEMG was utilized in several studies to precisely quantify fiber density, jitter, and blocking, offering high sensitivity for detecting subclinical NMJ transmission abnormalities and age-related changes in motor unit connectivity and muscle fiber reinnervation [24, 25]. Mechanomyographic (MMG) recordings, though less common, were used to assess muscle mechanical oscillations in response to voluntary and electrically evoked contractions, providing complementary insights into motor unit recruitment strategies and contractile behavior [26, 27]. These methodological approaches, ranging from non-invasive surface assessments to detailed intramuscular and single-fiber techniques, collectively enabled the multi-level characterization of neuromuscular decline associated with aging and sarcopenia.

### Age-related changes in muscle mass and performance

Across the included studies, both cross-sectional and longitudinal designs consistently demonstrated significant age-related reductions in muscle mass and strength. Lamoureux et al. observed a decline in maximal voluntary contraction (MVC), one-repetition maximum (1-RM) strength, and bone-free lean tissue (BFLT) mass in older adults compared to a younger-old cohort, with the most pronounced differences in lower-limb muscle groups [5]. Similarly, Piasecki et al. found that older males had significantly lower appendicular lean mass and quadriceps cross-sectional area (CSA), accompanied by decreased grip strength and knee extension MVC relative to young controls [19]. MRI-based studies further confirmed that muscle atrophy, particularly in the anterior thigh, is a hallmark of aging and sarcopenia progression [8].

In post-menopausal women, Willoughby et al. reported increased body fat, decreased muscle quality, and reduced relative strength compared to pre-menopausal women, despite similar fat-free mass, highlighting the impact of hormonal changes on muscle function [16]. Sarto et al. documented a progressive reduction in appendicular lean mass and quadriceps CSA across non-sarcopenic, pre-sarcopenic, and sarcopenic groups, underscoring the continuum of muscle loss with advancing sarcopenia [21].

### Motor unit remodeling and firing patterns

Advanced age is associated with altered motor unit behavior. Soderberg et al. (1991) [14] reported increased interspike intervals (ISI) in elderly subjects (64–82 years old, 110–117 ms) compared to young adults (23–35 years old, 88–97 ms), indicating slower firing rates and greater variability. Akataki et al. (2002) [27] further demonstrated that elderly men (69.8 ± 4.7 years old) showed distinct mechanomyographic (MMG) responses, with delayed and extended recruitment of slow-twitch MUs, delayed fast-twitch MU recruitment, and overall lower MMG and mean power frequency throughout submaximal force levels, reflecting a compensatory shift in recruitment strategy. Kent-Braun et al. [28] found no evidence of central activation failure in older adults during moderate fatigue, but baseline compound muscle action potential (CMAP) amplitudes were lower in older versus younger subjects, indicating reduced peripheral excitability. Older adults also displayed greater fatigue resistance, potentially due to metabolic adaptations. Watanabe et al. (2020) [17] found that MFGM supplementation during resistance training mitigated declines in MU firing rates, suggesting a potential neuromuscular benefit independent of muscle hypertrophy.

Kwon et al.’s (2014) study showed that age-related motor unit loss precedes clinically relevant muscle mass and functional loss [11]. Sarto et al.’s (2024) study showed that at different stages of human sarcopenia, surviving MUs in older adults (75.9 ± 4.7 years) are larger, and lower motor unit number estimate (iMUNE) in all older groups, confirming age-related MU loss. Increased complexity via near fiber EMG (phases, turns) was observed in older groups and reduced mean firing rate in sarcopenic group, and exhibits compensatory reinnervation of orphaned fibers [21]. This pattern is reinforced by findings of elevated near fiber jiggle and jitter (EMG markers of impaired transmission) in older and sarcopenic individuals. Importantly, most neuromuscular parameters did not differ between older individuals with or without sarcopenia.

Notably, motor unit loss is not uniformly distributed: distal muscles show greater loss than proximal ones, explaining differential strength loss patterns [11]. Even among highly trained master athletes (around 80 years old), significant MU loss and remodeling occur, challenging the notion that lifelong high-intensity exercise fully protects against age-related neuromuscular remodeling [10, 18].

Guo et al. (2025) [23] identified sex-based disparities in neuromuscular aging. Older females exhibited higher MU firing rates but significantly smaller muscle size and lower strength than males, suggesting females start from a lower functional baseline despite parallel decline trajectories. Sarto et al. [21] observed that most neuromuscular deficits (e.g., lower firing rates, increased NMJ transmission impairment, elevated MUP complexity) were present in all older groups regardless of sarcopenia stage, suggesting that the majority of age-related neuromuscular decline occurs before the clinical threshold of sarcopenia is reached. This highlights the “ageing health-sex paradox” where females live longer but with poorer neuromuscular health.

### Neuromuscular activation and aging

Neuromuscular activation was a key focus in several studies. Lamoureux et al. (2001) demonstrated that the old group (63.3 ± 2.6 years old) had a significantly higher percentage of peak integrated electromyographic activity compared with older group (75 ± 3.9 years old), indicating that older adults experience alterations in neuromuscular activation patterns [5]. Olmos et al. (2019) reported that middle-aged (45.1 ± 2.7 years) and older males (65.3 ± 3.2 years) exhibited similar plantarflexion peak torque (*p* > 0.05), but older males demonstrated 25% lower rate of torque development (RTD) (*p* < 0.01) and 18% reduced rate of EMG rise (RER) (*p* < 0.05), indicating impaired rapid neuromuscular activation [12]. These changes were also observed in the study of Piasecki et al. (2016), where older adults (71.4 ± 6.2 years old) showed larger MUPs and reduced firing rates, suggesting that aging affects the stability and efficiency of neuromuscular transmission. Also, Piasecki et al. (2016) reported that older adults exhibited a 35–50% reduction in motor unit number estimation (MUNE) (*p* < 0.001) compared to younger cohorts, reflecting progressive motor unit loss during aging.

Kwon et al. (2014) reported that older adults had greater EMG burst duration and amplitude during goal-directed tasks, indicating compensatory mechanisms in neuromuscular activation to maintain performance despite aging [11]. Additionally, Clark et al. (2011) found that the rate of EMG rise was positively associated with dynamic muscle performance, suggesting that slower neuromuscular activation rates in older adults could contribute to diminished functional strength and mobility [6]. The study of Sawers et al. (2017) showed that older females (≥ 65 years) who fell during laboratory-induced slips exhibited 40% longer EMG onset latencies in the biceps femoris long head (BFLH) and vastus lateralis (VL) compared to those who recovered (*p* < 0.01). Delayed activation in the leading/slip leg correlated with 50% greater slip distance (*r* = 0.62, *p* < 0.001) [13]. The findings from the study by Gilmore et al. (2017) further emphasized the importance of neuromuscular function, noting no significant differences in muscle thickness among pre-sarcopenic, sarcopenic, and severe sarcopenic groups. However, they highlighted that the voluntary activation capacity of dorsiflexion declined significantly with advanced sarcopenia [20]. This decline in voluntary activation was associated with decreased muscle strength and performance, reinforcing that neuromuscular control is a critical factor influencing mobility in older adults.

### Neuromuscular junction integrity in older adults

Research consistently demonstrates that NMJ integrity declines with age, and this deterioration is detectable through multiple biomarkers and electrophysiological parameters before overt muscle atrophy or functional impairment occurs. Several studies included in the document used both morphological and biochemical markers to quantify NMJ health in older populations, including specific patient age ranges and test values.

A prominent biomarker of NMJ degradation is the C-terminal agrin fragment (CAF), whose elevation signals increased breakdown of the NMJ. For example, in a cross-sectional study of pre- and post-menopausal women, post-menopausal subjects exhibited markedly higher CAF levels (3860.20 pg/mL) compared to pre-menopausal counterparts (1208.40 pg/mL), indicating accelerated NMJ degradation after menopause. This change was accompanied by increased neurotrypsin activity, disrupted acetylcholine receptor (AChR) clustering, and reduced motor unit activation, all pointing toward impaired NMJ transmission and recruitment patterns [16].

In older adults—both with and without clinically diagnosed sarcopenia—biochemical and electrophysiological evidence for NMJ instability is compelling. In Sarto et al.’s (2024) study of 88 older individuals (mean age 75.9 ± 4.7 years) and 42 young controls, older groups showed elevated near-fiber segment jitter (a measure of NMJ transmission instability), increased CAF, altered caveolin 3 expression, and a 3.8-fold higher prevalence of NCAM-positive fibers (indicative of denervation-reinnervation cycles) compared to young participants. Additionally, serum neurofilament light chain, a marker of axonal damage, was consistently elevated in the elderly, further supporting the presence of ongoing neurodegeneration [21]. Notably, these neuromuscular alterations were present even in non-sarcopenic older adults, suggesting that NMJ decline precedes overt functional loss.

The effects of inactivity on NMJ structure and function were highlighted in Motanova et al.’s (2024) study involving 10 older men (mean age 68.5 ± 2.6 years) subjected to 10 days of bed rest. Post-intervention assessments revealed a significant reduction in the overlap between presynaptic and postsynaptic terminals, decreased endplate occupancy (more denervated/partially denervated endplates), and enlarged AChR areas and perimeters. Functionally, there was a 9% rise in circulating CAF and significant increases in near-fiber jitter and jiggle, confirming functional impairment of the NMJ as a result of short-term disuse [22].

Reference data for NMJ parameters in the healthy elderly population are essential for distinguishing normal aging from pathology. Balci et al.’s (2025) study established normative values for single-fiber EMG parameters in the frontalis muscle of subjects aged 70–79 (mean 73.9 ± 1.7 years) and > 80 (mean 82.2 ± 1.2 years). The upper limits (95% confidence) for jitter were 40.4 μs in the 70–79 age group and 43.7 μs in those over 80, while fiber density limits were 1.90 and 2.14, respectively. Both parameters increased with age, reflecting compensatory reinnervation processes [24]. Finally, as sarcopenia progresses in adults aged 75–82, NMJ instability becomes more pronounced. In Gilmore et al.’s (2017) cohort categorized as pre-sarcopenic, sarcopenic, and severely sarcopenic, near-fiber jiggle and jitter rose by 31% and 43%, respectively, in those with severe sarcopenia compared to pre-sarcopenic individuals. However, motor unit number estimates did not significantly differ across groups, reinforcing the notion that NMJ transmission instability (rather than sheer motor unit loss) is a more sensitive marker of sarcopenia severity [20].

In summary, NMJ degradation and impaired transmission—as reflected by increased CAF, near-fiber jitter/jiggle, NCAM positivity, and neurofilament light chain—are early and measurable features of neuromuscular aging. These changes are accelerated by menopause and inactivity, can be detected before significant muscle loss occurs, and are crucial for early identification and intervention in age-related neuromuscular decline. Age-specific reference values for NMJ parameters further aid in distinguishing physiological aging from neuromuscular pathology.

### Functional implications and mobility

Neuromuscular changes correlate strongly with functional impairments. Clark et al. (2014) reported that rate of EMG rise independently predicted walking speed in older men but not women, highlighting sex-specific determinants of mobility [9]. Studies also showed that impaired neuromuscular activation rates are closely linked with decreased power and muscle performance, especially in mobility-limited older adults [12]. Despite preserved muscle size and strength, older males (65.3 ± 3.2 years old) showed significant decreases in peak power (PP) (*p* = 0.046, *d* = 0.79), rate of power development (RPD) (*p* = 0.026, *d* = 0.90), rate of torque development (RTD) (*p* = 0.022, *d* = 0.91), and rate of EMG rise (RER) (*p* = 0.010, *d* = 1.04) than middle-aged (45.1 ± 2.7 years old). Kwon et al. (2014) demonstrated that altered muscle activation patterns contribute to impaired goal-directed movement control in older adults, with differing neuromuscular parameters influencing upper and lower limb accuracy [11]. Longitudinal studies confirm that neuromuscular activation declines by ~ 9% per year, with a 28% overall decrease over three years, contributing to power loss before strength or mobility is detectably impaired [8].

The study by Motanova et al. (2025) revealed that short-term immobilization or bed rest (10 days) causes rapid and disproportionate declines in muscle strength (31%) relative to muscle size (15%), suppression of MU firing rates (8–11%), decreased MU potential size, and increased complexity—all confined to the immobilized limb in 10 older males (68.5 ± 2.6 years old) [29]. Bed rest in older adults also leads to morphological NMJ changes (reduced presynaptic/postsynaptic overlap, increased AChR area), elevated agrin fragments, and impaired transmission stability, underscoring the vulnerability of the aging NMJ to disuse.

### Effects of interventions: dietary supplementation and exercise on neuromuscular decline

Interventions aimed at improving neuromuscular performance have been shown to have significant effects, particularly in older adults who often face declines in muscle function and strength. Various studies have illustrated the potential benefits of both exercise and nutritional strategies on neuromuscular health. Watanabe et al. (2020) [17] reported that both older adults in the placebo and milk fat globule membrane (MFGM) (1 g/day) groups exhibited no significant differences in muscle mass or MVC after isometric knee extension resistance training (8 weeks, twice per week). However, the MFGM group showed increased firing rates of motor units after 2 weeks of intervention, suggesting that nutritional supplementation might enhance neuromuscular adaptations in older adults undergoing resistance training. In Soga et al.’s (2015) randomized, double-blind, placebo-controlled, crossover trial, 14 healthy Japanese adults aged 31–48 years received MFGM supplementation (1g daily, equivalent to 600mL whole milk) alongside twice weekly low-intensity cycling for 4 weeks, with a 4-week washout before crossover. Results showed that the MFGM group had a significant improvement in isokinetic leg extension strength compared to placebo, with surface EMG indicating a + 12.52% increase in RMS amplitude (versus a − 8.92% decrease in placebo; *p* = 0.028), suggesting enhanced motor unit recruitment. Notably, these strength gains occurred without changes in muscle mass, pointing to predominantly neurological rather than hypertrophic adaptations. Additionally, the MFGM group exhibited increases in RBC count and hemoglobin, with no adverse effects reported [30].

Additionally, the study by Power et al. (2016) on master athletes demonstrated that high-performing older adults (around 80 years old) maintained superior neuromuscular stability and muscle strength compared to their age-matched counterparts [18]. Higher performing older adults showed significantly higher dorsiflexion strength (both males and females) compared with age-matched controls. This finding indicates that continuous engagement in physical activity throughout life can effectively preserve neuromuscular function and mitigate the adverse effects of aging on muscle performance [10]. Moreover, resistance training has been shown to induce positive changes in NMJ health. Engaging in regular exercise, particularly resistance training, can counteract age-related declines in neuromuscular function by promoting NMJ plasticity and enhancing the capacity for motor unit recruitment [31–33]. This is critical for maintaining functional strength and mobility in older populations.

These findings underscore the importance of implementing targeted interventions that focus on exercise and nutrition to enhance neuromuscular performance. These interventions can ultimately help combat age-related muscle decline and improve the quality of life for older adults.

### Preclinical studies

#### Age-related changes in muscle mass and strength

Across multiple preclinical studies, aging is consistently associated with reductions in muscle mass and strength, although the pattern and severity vary depending on the muscle group, sex, and the intervention being tested. For instance, Padilla et al. (2021) [34] reported that absolute muscle mass was significantly lower in aged male rats compared to young males, while aged females showed a similar but less pronounced decrease relative to young females. When muscle mass was normalized to body mass, significant declines were observed across both sexes with age. Specifically, in this study, absolute muscle mass was significantly lower in aged males compared to young males, but not in aged females compared to young females. Additionally, significant reductions in muscle mass were observed when normalized to body mass and compared across all sexes.

Muscle strength, most often assessed via grip strength or direct measurement of tetanic/twitch force, also declined with age. In Padilla et al. (2021) [34], hindlimb grip testing revealed a significant loss of muscle strength (both absolute and normalized to body mass) between young and aged rats. Additionally, Yamaguchi et al. (2025) [35] found that after 2 months of intervention, grip strength was significantly lower in aged control mice (24–26 months) compared to both young (3–5 months) and endurance-trained aged mice, with the most marked deficits seen in hindlimb muscles (plantaris and soleus). In contrast, forelimb muscles showed greater resistance to age-related strength loss, as illustrated by the lack of significant differences in forelimb grip testing between young and aged animals. Pannérec et al. (2016) further highlighted muscle-specific vulnerability: hindlimb muscles such as the tibialis anterior (TA) and gastrocnemius exhibited up to ~ 50% atrophy by 24 months of age in rats, while forelimb muscles (e.g., biceps brachii) maintained stable muscle mass and fiber cross-sectional area even in old age, showing neither significant atrophy nor a shift in fiber types [36]. These studies indicate that aging leads to substantial losses in muscle mass and force production, especially in hindlimb muscles, whereas forelimb muscles and their associated nerves are relatively spared from age-related atrophy and weakness, possibly reflecting differences in muscle usage, innervation, or intrinsic susceptibility to sarcopenia.

### Morphological changes at the neuromuscular junction

Multiple studies report that aging is accompanied by pronounced morphological alterations at the NMJ. In general, old animals exhibit a progressive increase in NMJ fragmentation and denervation compared to their younger counterparts. For example, Chung et al. (2017) [37] examined C57BL/6 mice and found that postsynaptic motor endplates of 24-month-old animals were highly fragmented and disorganized, in contrast to the classic pretzel-shaped, well-organized AChR clusters seen in 3-month-old mice. The old mice also showed significantly lower presynaptic terminal coverage of postsynaptic areas and increased incidence of complete denervation (i.e., fibers with AChRs diffusely distributed along the sarcolemma without presynaptic apposition). Similarly, Padilla et al. (2021) [34] reported that aged rats (specifically, male and female rats aged 28–30 months) had significantly higher NMJ fragmentation, as well as increased denervation, compared to young (6–8 months) animals. The postsynaptic AChR clusters in aged rats were more fragmented, and there was a marked reduction in motor unit number estimation (MUNE) and increased jitter and blocking on single-fiber electromyography. Ham et al.’s (2020) study also showed that aged mice (30 months) exhibited 45% more fragmented [38] acetylcholine receptor (AChR) clusters (*p* < 0.001) and 30% reduced presynaptic terminal occupancy (*p* < 0.01) compared to young mice (8 months) [39].

In fast-twitch muscles, these age-related changes are particularly significant. Yamaguchi et al. (2025) [35] found in 24-month-old C57BL/6 J mice that the plantaris muscle (predominantly fast-twitch fibers) had a significantly higher proportion of denervated NMJs, reduced presynaptic terminal area, and decreased synaptic overlap compared to both 4-month-old and endurance-trained aged mice. Interestingly, denervation was more frequent in fast-twitch than slow-twitch fibers. Pannérec et al. (2016) [36] reported that in rats, NMJ fragmentation increased progressively with age in hindlimb muscles such as the extensor digitorum longus (EDL) and tibialis anterior (TA), reaching up to 70% fragmentation by 24 months. In contrast, forelimb muscles (e.g., biceps brachii), which are more resistant to sarcopenia, maintained stable NMJ morphology and did not show significant fragmentation, highlighting regional and muscle-type specificity in age-related NMJ degeneration.

Consistent results were also observed in mouse models of sarcopenia. Zhang et al. (2024) [40] found that NMJ fragmentation and degeneration were prominent in skeletal muscles of both aged mice (24 months) and muscle-specific Sirt6 knockout mice, with postsynaptic AChR clusters displaying discontinuity and degeneration. These morphological changes were linked to impaired neuromuscular transmission. Zhao et al. (2018) [41] demonstrated that in 24-month-old C57BL/6 J mice, there was a significant increase in fragmented and denervated NMJs, with only about 50% of endplates fully innervated, compared to nearly complete innervation in 3-month-old mice. Blasco et al. (2020) [42] examined various hindlimb muscles in old C57BL/6 J mice (27–29 months) and observed extensive NMJ remodeling, including partial or complete denervation, polyinnervation, increased terminal branching, sprouting, and larger, fragmented endplates. Chung et al. (2017) [37] also reported that, despite the increased postsynaptic area in old mice, the occupancy of this area by presynaptic terminals was significantly reduced, and complete denervation (characterized by diffuse AChR distribution without presynaptic apposition) was common.

### Electrophysiological assessment of motor unit and NMJ function

Electrophysiological studies in preclinical models have provided sensitive and complementary insights into NMJ integrity and function during aging.

#### Single-fiber electromyography and jitter analysis

Single-fiber EMG (SFEMG) is a gold standard for quantifying NMJ transmission variability (“jitter”) at the level of individual muscle fibers. Age-related increases in SFEMG jitter and blocking—a measure of transmission instability and failure—have been consistently documented in aged rodents. Chung et al. (2017, 2018) [37, 43] studied C57BL/6 mice (young: 4–6 months; old: 24–28 months), recording from hindlimb muscles. They found significantly increased SFEMG jitter and blocking in old mice compared to young, even before overt muscle atrophy or weakness was apparent. Chugh et al. (2020) [44] extended these findings in C57BL/6 mice aged 20–29 months, showing that jitter and blocking rise sharply at advanced ages, correlating with frailty and reduced muscle contractility. Padilla et al. (2021) [34] in rats (young: ~ 6 months, aged: ~ 22–24 months) observed higher SFEMG jitter/blocking in aged hindlimb and forelimb muscles, with strong correlations to muscle weight and MUNE. Padilla et al. (2024) [45] confirmed these findings in aged C57BL/6 mice (~ 24 months), noting the utility of SFEMG for detecting early NMJ dysfunction. Importantly, increased SFEMG jitter showed a strong inverse correlation with grip strength and spontaneous standing activity in aging mice, whereas compound muscle action potential (CMAP) amplitude did not, demonstrating SFEMG’s sensitivity to early NMJ changes. Jitter and blocking also correlated significantly with muscle weight, MUNE, and CMAP decrements during repetitive stimulation.

#### Compound muscle action potentials

CMAP amplitude, elicited by supramaximal stimulation of the motor nerve, reflects the global efficacy of neuromuscular transmission. While CMAP amplitude declines with advanced NMJ dysfunction, it is less sensitive to early changes and does not always correlate with initial functional decline or muscle weakness. Iyer et al. (2021) [46] used CMAP recordings in gastrocnemius and soleus of aged mice (20–24 months) and found a progressive loss of amplitude with aging, with some preservation in follistatin-treated groups. Chugh et al. (2020) [44] (20–29 months, C57BL/6) demonstrated CMAP amplitude reduction predominantly during high-frequency repetitive nerve stimulation (RNS), indicating a compromised safety factor of transmission in advanced age. RNS is especially useful for revealing NMJ transmission fatigue in aged muscles. Padilla et al. (2021) [34] in rats, and Padilla et al. (2024) [45] in mice, observed significant decrements in CMAP amplitude during repetitive trains in aged animals, closely correlating with muscle mass and physical strength. Zhang et al. (2024) [40], Zhao et al. (2018) [41], and Blasco et al. (2020) [42] observed similar age-related CMAP reductions in various mouse strains and muscles, including evidence from RNS and functional motor unit testing. Miyamaru et al. (2012) [47] and Ueta et al. (2020) [48] reported improved CMAP amplitudes following reinnervation or gene therapy in aged rat and mouse muscles, respectively (aged rats, ~ 26–30 months; aged mice, 28 months).

#### Motor unit number estimation and single motor unit potential analysis

MUNE, calculated from incremental CMAPs, quantifies the number of functional motor units and is a sensitive marker of age-related denervation. Chugh et al. (2020), Padilla et al. (2021, 2024), and Yamaguchi et al. (2025) (rodents aged 20–30 months) consistently found that MUNE declines with age in both hindlimb and forelimb muscles, correlating with muscle mass, grip strength, and SFEMG jitter/blocking [44]. Padilla et al. (2021) reported higher single motor unit potential (SMUP) amplitude in aged rats, consistent with collateral reinnervation by surviving motor axons. Yamaguchi et al. (2025) [35] also showed that MUNE drop is partially restored by endurance training.

#### Miniature endplate potentials/currents and endplate potentials

Intracellular microelectrode recordings of miniature endplate potentials (MEPPs), miniature endplate currents (MEPCs), and endplate potentials (EPPs) offer detailed pre- and postsynaptic functional assessment. Fahim et al. (1997) [49] reported that aged (28-month-old) unexercised mice exhibited a notable increase in nerve terminal junctional area, perimeter, and extension length compared to their younger (10-month-old) counterparts, suggesting that NMJs become larger and more complex with age. This morphological change was accompanied by functional alterations, including a 19% increase in miniature end-plate potential (MEPP) amplitude with aging, indicating an enhanced transmitter release capacity at NMJs. However, aged mice also experienced decreased MEPP frequency and a significant reduction in muscle strength, as evidenced by lower twitch tensions, particularly in unexercised groups. Kelly et al. (1986, 1987, 1983) (CFB-1 and CFW mouse strains, ages 10–31 months) [50–52] showed increased EPP amplitude and quantal content with age, with unchanged or reduced MEPP amplitude depending on muscle and age. These changes occurred before overt muscle atrophy. Wilson et al. (1988) stated that major changes in quantal content only occur between 28 and 42 days of age. After 42 days, increases are minor and statistically insignificant, indicating functional maturity [53]. Zhang et al. (2024) [40] (muscle-specific Sirt6 knockout mice, ages 18–24 months) and Zhao et al. (2018) [41] (aged mice 24 months) reported decreased MEPP amplitudes in aged or NMJ-compromised muscles, consistent with postsynaptic receptor loss. Alshuaib et al. (1991, 1990) (C57BL/6NNia mice, 10 vs. 28 months) [54, 55] and Smith et al. (1988) (rats, 8–28 months) [56] observed increased Ca^2+^-dependent quantal release and altered MEPP/EPP oscillatory properties in aged animals. The increase in quantal content is interpreted as a compensatory mechanism for morphological NMJ degeneration. Rosenheimer et al. (1985) [57] stated that assessed by MEPP, glucose uptake into nerve terminals varies by muscle fiber type, Alshuaib et al. (1990, 1991) demonstrated that increased Ca^2+^ influx at aged nerve terminals, prolonged post-tetanic potentiation (PTP), and altered oscillatory miniature excitatory postsynaptic potential (MEPP) frequency dynamics, indicating both compensatory plasticity and increased vulnerability to synaptic fatigue. Smith et al. (1988) (rats, diaphragm, 8–28 months) linked increased acetylcholinesterase activity and altered synaptic current kinetics to prolonged ACh actions and postsynaptic receptor field expansion in aged muscle.

#### Impact of exercise and nutrition supplement on muscle and NMJ adaptations

Exercise appears to have a dual effect on muscle and NMJ morphology, depending on the age of the subjects. The study by Fahim et al. (1997) has stated that in young mice (7 months old), endurance treadmill training (28 m/min for 60 min/day, 5 days/week for 12 weeks) led to hypertrophy of the NMJs, characterized by significantly larger nerve terminal areas and longer extension lengths. Conversely, in 25-month-old mice, the same endurance training resulted in smaller NMJ areas and shorter perimeters, indicating that while exercise can enhance NMJ morphology in younger muscles, it may affect the structure of NMJ in aged muscles[49]. Fahim et al. (1997) also noted that 12-week treadmill exercise improved the MEPP amplitude in young mice but decreased it in older mice. This suggests that the beneficial effects of exercise on NMJs may be age-dependent, with young NMJs responding positively to physical activity, whereas older NMJs show signs of deterioration. Regarding functional outcomes, exercise is associated with decreased MEPP frequencies in younger mice and increased frequencies in older mice, further demonstrating the complex interplay between age and exercise. The study by Yamaguchi [35] measured ankle plantar flexion torque evoked by nerve and muscle stimulations at physiologically relevant frequencies in young (5-month-old), aged control (24-month-old), and aged trained mice subjected to 2 months of endurance training. This study showed that ageing impairs neuromuscular transmission, especially during high-frequency stimulation, as shown by a reduced torque ratio (nerve stimulation torque to muscle stimulation torque) in aged mice compared to young and aged trained mice. The degree of pre- to post-synaptic overlap at the NMJ decreases with age and correlates positively with neuromuscular transmission efficiency. Age-related denervation occurs preferentially in fast-twitch muscle fiber, leading to a shift in myofiber composition toward a slower phenotype.

Chung et al. (2018) reinforced these findings, noting that increased SFEMG jitter levels in older animals (22–25 months old) correlated inversely with grip strength, thereby suggesting that neuromuscular transmission failure is a significant contributor to sarcopenia in aged populations [43]. This study highlighted that NMJ transmission deficits could precede observable declines in muscle strength, underlining the need for targeted interventions in the early stages of aging [35]. Endurance training partially prevents age-related denervation and the shift in myofiber composition, maintaining voluntary muscle strength. MUNE declines with age but is improved by endurance training.

Caloric restriction (40% reduction for 6–12 months) similarly preserved NMJ structure and maintained higher muscle contractile force compared to ad libitum–fed old mice. In Padilla et al.’s (2024) study [45], a ketogenic diet (KD; 90% kcal from fat administered to 18–24 month-old mice for 8–12 weeks) improved motor unit number estimation (MUNE increased by 20–30%), enhanced SFEMG stability (decreased mean jitter), and resulted in better grip strength and rotarod performance. In two studies targeting nutritional treatment [17, 58], mice receiving milk fat globule membrane (MFGM) supplementation (100 mg/kg/day) plus treadmill exercise displayed reduced NMJ fragmentation and improved motor coordination scores (balance beam and rotarod) compared to exercise or MFGM alone. Partial inhibition of class III PI3K VPS-34 (using SAR405) in 20–24-month-old mice and aged *C. elegans* improved NMJ transmission (increased quantal content by ~ 25%) and extended health span (median survival increased by 10–15%) [59]. Nerve-muscle pedicle implantation in denervated laryngeal muscles of 24-month-old rats promoted reinnervation and partial recovery of evoked EMG responses. These findings highlight that, even in advanced age, targeted molecular, nutritional, or regenerative strategies can preserve or restore NMJ function.

### Molecular and cellular mechanisms

At the molecular level, several key pathways have been implicated in age-related NMJ degeneration and associated motor decline. In aged mice (24 months) [40], muscle-specific deletion of Sirt6 led to accelerated NMJ degeneration and motor function decline. Supplementation with nicotinamide mononucleotide (NMN) preserved NMJ integrity and sustained grip strength (improvement of 20–25% over controls). Mechanistically, Sirt6 deficiency decreased the expression of Dystrophin at the synapse by failing to mono-ADP-ribosylate and degrade the transcriptional repressor YY1, leading to impaired postsynaptic assembly and NMJ maintenance. Importantly, supplementation with nicotinamide mononucleotide (NMN) in aged mice increased mono-ADP-ribosylation of YY1, restored Dystrophin levels, reduced NMJ fragmentation, and significantly improved CMAP amplitudes and physical performance measures, such as grip strength and rotarod latency, by 20–25% compared to controls. These beneficial effects of NMN were absent in Sirt6-deficient mice, highlighting the necessity of Sirt6 for NMN’s action.

In Drosophila [60], the small GTPase regulator Trio was shown to be critical for NMJ structural maintenance during aging. Loss of Trio resulted in a dramatic decline in synaptic Rac activity, leading to early synaptic bouton fragmentation, reduced NMJ size, and premature motor decline as measured by a decrease in climbing ability. Conversely, overexpression of either Drosophila or human Trio preserved NMJ structure—even in very old flies—and sustained high-frequency neurotransmission, delaying the onset of age-related motor deficits. Similary, overexpression of matrix metalloproteinase 1 (MMP1) [61] in motor neurons led to premature NMJ fragmentation and a reduction in neurotransmitter release (quantal content), causing reversible motor dysfunction, such as impaired climbing ability. This effect was counteracted by overexpression of the tissue inhibitor of metalloproteinases (dTIMP), which delayed age-related motor decline.

Therapeutic interventions targeting NMJ integrity also show promise in mammals. DOK7 gene therapy [48] delivered via AAV injection to 22–24-month-old mice robustly increased the postsynaptic area of acetylcholine receptor (AChR) clusters and presynaptic nerve terminal size, reduced the percentage of denervated NMJs, and led to significant improvements in neuromuscular transmission. Functionally, this was reflected in enhanced rotarod performance, with latency to fall increased by approximately 40% compared to aged controls. Importantly, these effects were achieved without inducing muscle hypertrophy, suggesting direct benefits on NMJ innervation and mechanical properties of muscle fibers.

Follistatin-induced muscle hypertrophy [46], achieved through AAV9-mediated gene delivery in 24-month-old mice, resulted in a 25% increase in muscle mass, significant improvement in twitch and tetanic torque, and enhanced NMJ innervation. These mice exhibited higher CMAP amplitudes and reduced jitter and blocking on single-fiber EMG, indicating preserved NMJ function and improved motor unit recruitment.

Another key molecular player is sarcoglycan alpha (SGα) [41]. In aged mice (24–28 months), transgenic overexpression of SGα in skeletal muscle stabilized NMJ structure by promoting the stability of the LRP4 protein, a critical NMJ organizer. This intervention increased the proportion of fully innervated endplates, decreased the rate of NMJ denervation by about 30% compared to controls, and improved AChR cluster intensity and muscle force. Mechanistically, SGα interacts with LRP4, protecting it from proteasomal degradation and thereby preserving agrin-LRP4-MuSK signaling essential for NMJ maintenance.

In summary, animal studies using a range of advanced ages (18–30 months for mice/rats and age-matched *Drosophila* models) consistently show that NMJ degeneration, impaired synaptic transmission, and motor decline are hallmarks of aging. However, interventions such as endurance training, caloric restriction, ketogenic diet, MFGM supplementation, gene therapy (DOK7, follistatin), NMN, and PI3K inhibition improve NMJ structure, EMG markers, and physical performance, supporting their translational potential for preserving neuromuscular health in late life.

## Discussion

The NMJ plays a crucial role in transmitting signals from motor neurons to muscle fibers, thereby impacting muscle function and overall physical performance. As highlighted in our systematic review, aging is associated with significant alterations in NMJ structure and function, which can contribute to the development of sarcopenia—a progressive decline in skeletal muscle mass and strength [2, 62]. This discussion aims to synthesize the findings of our review, emphasize the implications for aging populations, and propose directions for future research.

The evidence presented in this review indicates that older adults often experience a decline in neuromuscular activation and muscle performance, reinforcing the idea that NMJ integrity is vital for maintaining physical capabilities. For instance, studies by Lamoureux et al. (2001) and Piasecki et al. (2016) demonstrated that older adults exhibited impaired neuromuscular activation patterns, characterized by larger motor unit potentials (MUPs) and reduced firing rates. These findings suggest that as individuals age, there is a remodeling of motor units, which may lead to decreased muscle performance and increased susceptibility to mobility limitations. The relationship between NMJ dysfunction and muscle performance is further emphasized by Clark et al. (2011, 2013, 2014), who established a significant correlation between voluntary neuromuscular activation and muscle output, particularly among older adults with mobility challenges.

While exercise plays a critical role in maintaining NMJ integrity, understanding the underlying mechanisms of NMJ dysfunction is equally important. Our review highlights age-related changes, such as axonal degeneration and NMJ innervation loss, which contribute to the decline in muscle function. The study of Chung et al. (2017) underscores the importance of investigating these mechanisms, as they provide insights for developing targeted interventions. For example, therapies aimed at enhancing NMJ stability and integrity could combat the age-associated decline in muscle performance. The mechanisms include mitochondrial dysfunction [63], where impaired mitochondrial transport and function result in energy deficits and increased oxidative stress [64, 65]. Additionally, an inflammatory response occurs, characterized by the breakdown of the blood–brain barrier and infiltration of reactive glial cells to aid in removing axonal debris [66, 67]. Dying-back degeneration is also significant, particularly in chronic neurodegenerative diseases, where axons degenerate from the synaptic regions towards the cell body. This process involves microtubule disassembly, leading to axonal swelling and fragmentation [68], and impaired axonal transport, which causes defective transport of essential proteins and organelles that contribute to axonal degeneration. Furthermore, reduced acetylcholine release can occur, impairing synaptic function, while loss of motor neurons reduces the number of motor units, leading to muscle fiber denervation [69, 70].

Furthermore, our findings suggest a complex interrelationship between NMJ dysfunction and the multifactual mechanisms of sarcopenia. The study conducted by Chugh et al. (2020) elucidates a significant correlation between increased SFEMG jitter and blocking with muscle weight, thereby highlighting NMJ transmission failures as a potential primary mechanism contributing to age-related muscle dysfunction [44]. SFEMG serves to quantify the temporal variability (jitter) and instances of action potential blocking within individual muscle fibers. An escalation in both jitter and blocking indicates compromised NMJ transmission, which is closely associated with muscle weakness and a decrease in muscle mass. Also, electrophysiological techniques, including MUP analysis, can detect NMJ alterations and provide insights into the extent of neuromuscular impairment at different stages of sarcopenia [21].

In evaluating the diagnostic efficacy of NMJ assessments [71] relative to other established tools for diagnosing sarcopenia, such as imaging modalities (e.g., magnetic resonance imaging[72], dual-energy X-ray absorptiometry [1]) and functional evaluations (e.g., grip strength, gait speed), it is important to conduct a comparative analysis. Electrophysiological methods reveal that neuromuscular aging involves early and progressive NMJ transmission deficits, often preceding or occurring in parallel with morphological degeneration. In clinical studies, while surface EMG is ideal for large-scale, non-invasive studies of muscle activation, needle EMG and especially SFEMG are superior for detecting early and subtle NMJ transmission defects. The choice of EMG modality should thus be guided by the specific research or clinical objective: initial screening and population studies may rely on surface EMG, whereas detailed pathophysiological investigations and early diagnosis of NMJ dysfunction necessitate the use of needle EMG or SFEMG. Multi-channel and high-density EMG represent promising advancements, offering the potential for non-invasive yet highly sensitive NMJ assessment in future research and clinical practice. In our review, we also discussed that SFEMG jitter/blocking and MEPP/EPP analyses are the most sensitive and mechanistically informative for detecting early and subclinical NMJ dysfunction, while CMAP and MUNE provide integrative, whole-muscle assessments. RNS is valuable for assessing fatigability, a clinically relevant phenotype.

The complex interplay between aging, exercise, and NMJ function also necessitates a nuanced approach to rehabilitation. As noted throughout our review, the effects of exercise on NMJs can be age-dependent, with younger NMJs responding positively to physical activity [73]. In comparison, older NMJs may exhibit signs of deterioration. Exercise has been shown to promote NMJ hypertrophy in younger individuals, enhancing synaptic stability and function. However, in older adults, the response can be more variable, with some studies indicating NMJ compression and reduced efficacy of synaptic transmission [73, 74]. This finding suggests that rehabilitation strategies should be tailored to the individual’s age, physical capabilities, and health status. Implementing personalized exercise programs that account for these factors could enhance the effectiveness of interventions aimed at preserving NMJ function and muscle performance in older adults. For instance, endurance training has been found to have a more pronounced effect on NMJ structural remodeling, particularly in fast-twitch muscle fibers [73, 75]. Studies have shown that endurance exercise leads to significant changes in the morphology of NMJs, including increased endplate area and dispersion in fast-twitch fibers. This remodeling enhances synaptic stability and function, which is crucial for maintaining muscle performance [76]. Additionally, endurance training promotes the renewal of synaptic structures in oxidative and oxidative-glycolytic muscle fibers, ensuring efficient neuromuscular transmission. These adaptations highlight the importance of tailored exercise programs in preserving NMJ health and muscle function, especially in aging populations [77, 78].

Regular exercise, particularly resistance training, has been shown to influence NMJ health and functionality positively. In clinical trials, Watanabe et al. (2020) found that nutritional supplementation, such as milk fat globule membrane and resistance training, enhanced motor unit firing rates in older adults. This suggests that combining nutritional strategies with exercise regimens may be a powerful approach to mitigate age-related declines in NMJ function. For instance, omega-3 fatty acids in fish oil have anti-inflammatory properties that support NMJ integrity, while antioxidants like vitamins C and E protect against oxidative stress-induced damage [79]. When these dietary components are paired with resistance training, which promotes muscle hypertrophy and NMJ remodeling, the benefits are amplified [80]. Additionally, protein-rich diets, including leucine, stimulate muscle protein synthesis and support NMJ stability [81]. Aerobic exercise, on the other hand, enhances mitochondrial function and increases blood flow [82], delivering essential nutrients to the NMJs. Combining these dietary and exercise interventions creates a robust environment for maintaining NMJ health, potentially delaying the onset of age-related neuromuscular decline and improving muscle function in older adults. For animal studies, across a wide range of animal models and ages—including mice and rats aged 18–30 months, and age-matched Drosophila—consistent evidence indicates that NMJ degeneration, impaired synaptic transmission, and progressive motor decline are hallmarks of natural aging. Notably, a variety of interventions have demonstrated efficacy in ameliorating these age-related changes. For instance, endurance exercise and caloric restriction can reduce NMJ fragmentation and denervation, improve grip strength, and enhance EMG markers. Ketogenic diet feeding in aged mice led to improved grip strength, rotarod performance, and increased MUNE, indicating preserved motor unit connectivity. Milk fat globule membrane (MFGM) supplementation combined with exercise preserved NMJ morphology, reduced denervation, and maintained peripheral nerve structure and conduction velocity. Additionally, gene therapies (such as DOK7 or follistatin), NMN supplementation, and PI3K (VPS-34) inhibition have all improved NMJ structure and function, as evidenced by increased CMAP amplitudes, reduced jitter and blocking on EMG, and better physical performance in aged animals.

Recent advancements in biotechnology and artificial intelligence (AI) are poised to revolutionize the understanding and management of NMJ dysfunction in aging and sarcopenia. High-resolution NMJ profiling, facilitated by state-of-the-art imaging modalities and molecular techniques, now allows for unprecedented visualization and quantification of NMJ morphology and synaptic alterations in both animal models and human tissue. Techniques such as super-resolution microscopy, single-nucleus RNA sequencing, and multiplex immunohistochemistry have enabled detailed mapping of NMJ architecture, receptor distribution, and molecular changes associated with aging and disease [80]. For example, the use of single-fiber and near-fiber EMG in combination with molecular biomarkers, such as C-terminal agrin fragment (CAF) and neurofilament light chain, offers a comprehensive approach to assess NMJ stability and degeneration in vivo [83]. Artificial intelligence, particularly machine learning algorithms, can further enhance the utility of these high-resolution datasets by enabling automated NMJ segmentation, quantification, and pattern recognition [84]. AI-driven image analysis tools can detect subtle morphological changes that may precede overt neuromuscular impairment, supporting early diagnosis and longitudinal monitoring. Moreover, predictive modeling using AI can integrate multi-omic, electrophysiological, and clinical data to forecast individual risk trajectories for sarcopenia or response to specific interventions. Such models hold promise for identifying novel biomarkers, stratifying patients for tailored interventions, and optimizing clinical trial design by predicting responders to therapies targeting NMJ preservation. Despite these promising developments, challenges remain. Standardization of data acquisition, validation of AI algorithms across diverse populations, and integration of multi-modal datasets require collaborative efforts among clinicians, biologists, and data scientists. Ethical considerations regarding data privacy and algorithmic transparency must also be addressed to ensure equitable implementation in clinical settings.

Despite the promising findings presented in our review, it is essential to acknowledge the limitations of the existing literature. Many studies included in our analysis were conducted with small sample sizes or utilized animal models, which may not fully represent the complexities of NMJ dysfunction in human aging. Despite significant advances in the understanding of NMJ dysfunction in aging and sarcopenia, several critical gaps remain that warrant focused investigation. Future research should prioritize the refinement of sensitive and specific biomarkers—including electrophysiological, biochemical, and imaging markers—to enable earlier detection of NMJ dysfunction before the onset of overt muscle atrophy and physical decline. Mechanistic studies are necessary to elucidate the molecular and cellular processes underlying NMJ deterioration, including mitochondrial deficits, inflammatory pathways, and impairments in axonal transport, thereby uncovering novel therapeutic targets. Additionally, large-scale and longitudinal human studies are essential to clarify the temporal progression and causal relationship between NMJ changes and sarcopenia, as much of the current literature is based on animal models or small human cohorts. Future directions should also explore the synergistic effects of combined interventions—including nutritional, pharmacological, and exercise-based strategies—while considering personalized approaches that account for individual factors like age, sex, genetics, and lifestyle. Innovative therapies, such as gene therapy, regenerative medicine, and new pharmacological agents aimed at preserving NMJ integrity, must be rigorously evaluated in both preclinical and clinical settings. Finally, bridging the gap between basic science discoveries and clinical application remains a priority, ensuring that advancements in NMJ preservation can be effectively translated to improve outcomes in older adults at risk of sarcopenia. By addressing these knowledge gaps, future research can accelerate the development and implementation of novel strategies to prevent and treat sarcopenia, ultimately promoting healthier aging in the population.
